# Grading disorder severity and averted burden by access to treatment within the GBD framework: a case study with anxiety disorders

**DOI:** 10.1016/S2215-0366(23)00037-8

**Published:** 2023-04

**Authors:** Damian F Santomauro, Caroline Purcell, Harvey A Whiteford, Alize J Ferrari, Theo Vos

**Affiliations:** a School of Public Health, University of Queensland, Herston, Queensland, Australia; b Queensland Centre for Mental Health Research, Wacol, Queensland, Australia; c Institute for Health Metrics and Evaluation, University of Washington, Seattle, Washington, USA

## Abstract

**Background:**

The Global Burden of Disease Study (GBD) estimates burden by cause with major relevance for resource allocators globally. Non-fatal burden estimates are influenced by disorder severity. However, for many disorders, global severity is sourced from a single high-income country survey. We aimed to estimate severity distributions that vary by Healthcare Access Quality Index (HAQI) using anxiety disorders as a case study and present the usefulness of this method in simulating averted and avoidable burden globally.

**Methods:**

In this case study, we estimated treatment use among respondents with anxiety disorder in the 1997 Australian National Survey of Mental Health and Wellbeing (NSMHWB), the source used to estimate severity of anxiety disorders in GBD. Treatment effects were sourced from the Cochrane Database of Systematic Reviews and pooled via network meta-analysis. Severity distribution was established via a meta-regression of their disability weights, derived from 12-item short form survey scores. We simulated the shift in severity across scenarios without access to treatment and with full access to optimal treatment (cognitive behavioural therapy and antidepressants). We interpolated this shift linearly along the HAQI, extrapolated country-specific severity from HAQI scores, and calculated averted and avoidable burden.

**Findings:**

The database review sourced 56 reviews, of which eight were eligible for inclusion. These eight reviews reported on 156 randomised controlled trials, with 194 treatment effects. Respondents to the 1997 NSMHWB consisted of 5936 women (55·8%) and 4705 (44·2%) men aged 18 years or older (mean age and ethnicity data not available). The survey-weighted treatment effect size was –0·28 (95% uncertainty interval –0·45 to –0·12). The pooled treatment effect for full coverage optimal treatment was –1·07 (–1·47 to –0·64). The sequela-weighted disability weight among people with anxiety disorder in the NSMHWB was 0·141 (0·042 to 0·275). The estimated disability weight was 0·188 (0·070 to 0·341) after removing the benefits of treatment and 0·056 (0·013 to 0·140) after providing all people with anxiety disorder access to optimal treatment. Globally, 12·5% (4·6 to 21·5) of anxiety disorder burden was averted because of available treatment. However, 71·1% (46·2 to 87·6) of global anxiety disorder burden could be averted if all people with anxiety disorders had access to optimal treatment.

**Interpretation:**

Because it is based on guidance from a single survey done in one high-income country, the burden of anxiety disorders in low-income and middle-income countries is probably underestimated by GBD. Despite the availability of effective treatments, low use of these treatments means that most burden is still avoidable. Most of the burden could be averted if all people with anxiety disorders had access to optimal treatment, highlighting the importance of public promotion and referral pathways of treatment for anxiety disorders. Location-specific severity distributions in GBD would greatly increase precision in burden estimates and highlight avertable burden to clinicians, public health practitioners, and policy makers.

**Funding:**

Queensland Health and Bill & Melinda Gates Foundation.

## Introduction

The Global Burden of Diseases, Injuries, and Risk Factors Study (GBD) 2019 is the largest scientific effort to estimate the prevalence and health burden of disorders, diseases, injuries, and risk factors.^1^ The results of GBD are used by policy makers, service planners, resource allocators, and researchers worldwide. GBD quantifies health burden via the disability-adjusted life-year (DALY), which represents the years of healthy life lost due to a disorder or injury. The DALY is the sum of the years lived with disability (YLDs), representing non-fatal burden, and the years of life lost, representing fatal burden. This allows for comparison and ranking of all causes of burden.

In GBD 2019, the estimation of YLDs has three primary inputs: prevalence, severity distribution, and disability weights.^1^ The prevalence of the disorder is estimated by age, sex, region, and year. If the disorder occurs along a spectrum of severity (eg, Crohn's disease or major depressive disorder), the prevalence estimate is split into sequela-specific prevalence. Sequela-specific YLDs are estimated by multiplying a sequela-specific prevalence by its respective disability weight. Disability weights represent the health loss from a sequela on a scale of 0 (no health loss) to 1 (death). Finally, the YLDs for the disorder are the sum of the sequela-specific YLDs. Most causes of burden in GBD 2019 had acceptable numbers of data sources informing prevalence spanning many geographical regions. Estimated disability weights were consistent across several geographical regions.^2^, ^3^ However, for many causes of burden, the severity distribution used to split disorder prevalence across the globe into sequela-specific prevalence is estimated from a single survey from the USA or Australia.^4^ This paucity of data is because the severity distribution used in the estimation of burden of a disorder should reflect the disability specific to the disorder, which requires case-level data on both cumulative disability and the disorder's comorbidities. Other sources of severity considered for mental disorders in GBD, such as distributions provided by the World Mental Health Survey study group,^5^ are trans-diagnostic and tend to over-represent the proportion of people with severe mental disorder for the purposes of burden estimation.^6^ Nonetheless, the approach used in GBD 2019 assumed that the severity distribution of many disorders across the globe mirrors the severity distribution observed in a single high-income country. This assumption is questionable, because the severity distribution might be less severe in countries with higher quality health care and greater access to effective treatments.^7^ Therefore, GBD could be underestimating burden in countries with lower quality health systems or scarce access to care.

**Research in context**

**Evidence before this study**

Many non-fatal causes of disease burden are responsive to health-care interventions; however, the Global Burden of Diseases, Injuries, and Risk Factors Study (GBD) 2019 did not consider the effect of health-care access or treatment on disease severity. We searched PubMed for articles published between Jan 1, 1996, and Jan 21, 2022, that focused on estimating the severity distribution for anxiety disorders within a global burden of disease framework. We used the following search terms, without any further restrictions: ((anxiety[Title/Abstract]) AND (disorder*[Title/Abstract])) AND ((((((“disability weights”[Title/Abstract]) OR (“disability weight”[Title/Abstract])) OR (“health burden”[Title/Abstract])) OR (“burden of disease”[Title/Abstract])) OR (“severity distributions”[Title/Abstract])) OR (“severity Distribution”[Title/Abstract])). Our search produced 354 studies, of which one seemed related to our research aim. This study, published in 2004, estimated the averted and avoidable burden of mental disorders in Australia and the cost-effectiveness of avoiding mental disorder burden. However, no studies mirrored the methods in our study or simulated anxiety disorder severity by global region.

**Added value of this study**

We present a novel method to estimate a severity distribution for anxiety disorders that varies by region and health-care access. We also present additional usefulness of this method in simulating the global proportions of averted and avoidable burden due to anxiety disorders.

**Implications of all the available evidence**

Data from a mental health survey done in Australia has informed the severity distribution for anxiety disorders in GBD since GBD 2010. This underestimated the severity and burden of anxiety disorders in low-income and middle-income countries as it assumed the global severity distribution of anxiety disorders mirrored the severity distribution from one high-income country. The addition of region-specific severity distributions for anxiety disorders greatly increases the precision in burden estimates for non-high-income countries and potentially shifts the burden rankings for anxiety disorders for these regions. Furthermore, we distinguish the non-fatal burden of anxiety disorders that have been averted from the proportion that could be avoided and that which cannot be treated with existing treatments, thereby providing a roadmap for clinicians, public health practitioners, and policy makers to translate GBD findings into actionable results. This method could also be applied to other causes of burden in GBD to increase the precision of burden estimates and aid resource allocation by avoidable burden.

We aimed to develop a novel method to estimate region-specific severity distributions that vary by health-care access quality, using anxiety disorders as a case study. Anxiety disorders are characterised by intense and persistent feelings of fear and distress, often accompanied by behavioural disturbances and physiological symptoms. GBD 2019 reported that anxiety disorders were the 8th most common cause of YLDs and 24th most common cause of DALYs worldwide.^1^, ^8^ Anxiety disorder burden per person has remained stable since 1990 despite availability of effective treatments, because the prevalence of anxiety disorders remained unchanged and the same severity distribution was applied across geographical regions and time. Mood and anxiety disorder prevalence in high-income countries has not decreased as anticipated with improved treatment provision,^9^ and it seems intuitive that treatments would affect the severity of disorders before the prevalence. Therefore, any benefit of increased treatment coverage for anxiety disorders is unlikely to be reflected in GBD 2019, making anxiety disorders a good candidate for case study estimating severity by health-care access and quality.^10^

We also analyse the usefulness of estimating severity distributions that vary by health-care access quality in simulating the averted and avoidable burden of anxiety disorders by GBD super-region and globally for 2019. This provides insight into the effect of treatment availability in alleviating burden due to mental disorders.^11^ Previous work by Andrews and colleagues explored averted and avoidable burden of anxiety disorders in the 1997 Australian National Survey of Mental Health and Wellbeing (NSMHWB),^12^ but did not extrapolate this method globally or use GBD methods of estimating severity. GBD provides a snapshot for policy makers and service planners to highlight disorders causing the most burden for their given region. However, GBD does not provide any indication of which disorders should be prioritised on the basis of avoidable burden. Highly burdensome but untreatable disorders might garner more attention than less burdensome but treatable disorders. By breaking down non-fatal burden components into burden that has been averted, burden that could be avoided, and burden that cannot be affected by existing treatments, we aimed to provide a roadmap for clinicians, public health practitioners, and policy makers to translate GBD findings into actionable results.

## Methods

This study complies with the Guidelines for Accurate and Transparent Health Estimates Reporting recommendations (appendix pp 12–13).^13^ A graphical overview of the method is presented in the appendix (p 8). To summarise, data on treatment effectiveness were sourced from the Cochrane Library of Systematic Reviews and analysed in a network meta-regression to estimate pooled effect sizes by treatment. Coverage of these treatments was then estimated from the 1997 NSMHWB^14^ and used to weight the pooled treatment effect sizes into an overall coverage-adjusted treatment effect for anxiety disorders in the 1997 NSMHWB. The severity distribution of anxiety disorders in the 1997 NSMHWB was then estimated via a meta-regression of their disability weights (derived from their 12-item short form survey [SF-12] scores)^15^ controlling for comorbid conditions. The anxiety disorder-specific disability weights in the 1997 NSMHWB were then shifted on the basis of the coverage-adjusted treatment effect to reflect their likely disability weight if respondents with anxiety disorders had no access to treatment. This provided severity distributions between two anchor points: during the 1997 NSMHWB with its respective treatment access and in a scenario with no access to treatment. Coverage of minimally adequate treatment of anxiety disorders from the World Mental Health Surveys^16^ was then regressed against the Healthcare Access Quality Index^10^ (HAQI) to determine the relationship between treatment coverage and the HAQI, and the likely HAQI score where there would be no access to treatment. The severity distribution estimated in the 1997 NSMHWB and a scenario with no access to treatment was then linearly interpolated between the HAQI in Australia in 1997 and the likely HAQI score where there would be no access to treatment. This provided severity distributions for every corresponding HAQI score and in turn every country in GBD 2019. Location-specific YLDs were then estimated by use of the location-specific severity distributions and location-specific anxiety disorder prevalence from GBD 2019. All analyses were done with R (version 3.6.3). This process was done as a Bayesian Markov chain Monte Carlo method across 1000 samples from the posterior distribution of each step in the estimation process (eg, disability weights or treatment effects). The 95% uncertainty intervals (UIs) of all results were the 25th and 975th ranked values of the resulting distributions. Severity and avertable burden were estimated across sexes and ages in 2019 and followed the GBD location hierarchy.^1^

### Estimation of treatment effects

Data on the efficacy of health-care interventions were sourced from the Cochrane Database of Systematic Reviews. A title and abstract keyword search was done for anxiety disorders (“anxiety disorder” OR “anxiety disorders”) on April 27, 2022. Titles and abstracts were screened to determine which reviews to include. Reviews that did not assess an intervention for anxiety disorders; focused on non-standard or not recommended interventions (eg, valerian and antipsychotics); or focused on interventions for children, adolescents, or non-representative subpopulations (eg, women with breast cancer) were excluded. All remaining reviews were full-text screened and data for studies with outcomes relevant to disorder severity on a continuous scale were extracted. Data on intervention type, comparison type, follow-up duration, sample size, outcome mean, and outcome SD were extracted from each review.

Interventions were grouped into distinct classes as reported in the Cochrane reviews (cognitive therapy, behavioural therapy, cognitive behaviour therapy [CBT], psychodynamic therapy, supportive therapy, and antidepressants). The reference category was treatment as usual, waiting list, or placebo treatment.

The main outcomes assessed were symptom severity measured on a symptom scale. Efficacy was defined in terms of the standardised mean difference (SMD; ie, the difference in the scale mean values expressed as a fraction of the SD) in outcome measure between the intervention and comparison groups. The effect sizes for the longest follow-up per trial reported in each review were used in the analysis.

The SMD for each intervention versus comparison pair was estimated as the difference in the observed mean values divided by the pooled SD of the two groups using the escalc function in the metafor^17^ package in R. SMDs were then pooled using Meta Regression: Bayesian, Regularized, Trimmed (MR-BRT).^18^ We quantified the between-study heterogeneity of the SMDs and re-incorporated this variation into the estimated effect. The tool also allows for a trimmed maximum likelihood estimator, which excludes outlier effects based on their contributions to the likelihood function and trimming was set to 10% to maximise robustness of the effect sizes.^18^, ^19^ Dummy variables were created for each intervention type, with the exception of CBT (111 studies) for which the treatment effects were flagged on both the behavioural therapy and cognitive therapy dummy variables. This approach was used to maximise parsimony while incorporating the few studies that focused on either cognitive (two studies) or behavioural therapies (three studies). Data were analysed as a network to synthesise all available information across direct and indirect comparisons of each intervention class compared with the reference group (usual medical care, waiting list, or placebo).^20^

### Adjustment of treatment effects for treatment use

Treatment use and a cumulative treatment effect were estimated for respondents meeting criteria for anxiety disorders within the past 30 days in the 1997 NSMHWB (appendix p 3). The 1997 NSMHWB was a population-representative household survey in which 5936 women and 4705 men aged 18 years or older were interviewed via the Composite International Diagnostic Interview 3.0 to diagnose mental disorders. Respondents of the 1997 NSMHWB were asked about their service use over the past 12 months and were considered to have received CBT, other psychotherapy, or counselling if they reported that they undertook six or more sessions of that therapy delivered by a psychiatrist, psychologist, or other professionals specialising in mental health. Six or more sessions was chosen from the available session count categories in the 1997 NSMHWB anonymised unit record data (ie, one, two, three, four, five, six to ten, more than ten) that best aligned with definitions of minimally adequate treatment (ie, eight or more sessions).^16^ To align treatment data from the 1997 NSMHWB survey with the treatment effects from the network meta-analysis, other psychotherapy was assigned the treatment effect for psychodynamic therapy (as this was the only other psychotherapy treatment effect), and counselling was assigned the treatment effect for supportive therapy. Respondents also reported whether they took medication for mental health problems within the past 12 months and medication was assigned the treatment effect for antidepressants. We calculated the pooled mean treatment effect for all anxiety disorders (accounting for the complex survey design of the 1997 NSMHWB via the survey package in R^21^) and this was considered the coverage-adjusted treatment effect for Australia in 1997. A treatment effect for a full coverage optimal treatment scenario was also estimated for anxiety disorders. The optimal treatment effect size was the combination of the CBT treatment effect and the antidepressant treatment effect (as per Royal Australian and New Zealand College of Psychiatrists guidelines^22^) and coverage was assumed to be 100% for the full coverage optimal treatment scenario. Because disability weights are floored at zero, we assumed that patients requiring either CBT or antidepressants (not both) would receive the treatment necessary for their amount of disability in the full coverage optimal treatment scenario (appendix p 4).

### Severity estimation for anxiety disorders in GBD

We followed the same process to estimate the severity distribution for anxiety disorders in GBD 2019, which has been discussed elsewhere and is summarised here.^1^, ^4^ Since GBD 2010,^23^ the 1997 Australian NSMHWB^14^ has been used to inform the severity distribution for anxiety disorders in GBD. Respondents meeting criteria for anxiety disorders within the past 30 days were considered for this analysis to best align with the point prevalence of anxiety disorders estimated for GBD 2019. Respondents were also asked about their physical health conditions and functional health status as measured by the SF-12 scores. For our study, the composite SF-12 scores of each respondent in the NSMHWB were mapped to disability weights by use of an updated method that addressed limitations of the approach used in GBD studies from 2013 to 2019 (appendix pp 5–6).^1^, ^4^ This method estimates a cumulative disability weight for each respondent attributable to all conditions including both anxiety disorders and comorbid conditions.

Once NSMHWB respondents had been assigned cumulative disability weights, binary variables were created to represent the presence or absence of each health condition for each respondent.^1^, ^4^ Respondents’ disability weights were logit-transformed and regressed onto health conditions via MR-BRT^18^ to incorporate the uncertainty in respondents’ disability weights with fixed effects on each health condition and a random effect on respondent. This model was used to estimate the cumulative disability weights of all comorbid conditions for each respondent with an anxiety disorder.

This disability weight of the comorbid conditions was then removed from the respondents’ observed disability weight via the formula below to estimate the comorbidity-corrected disability weight for every patient with anxiety disorder in the 1997 NSMHWB, wherein DW represents disability weight.

$$DW_{anxiety\;disorders}=1-\frac{\text{1}-DW_{cumulative}}{\text{1}-DW_{comorbidities}}$$

Participants with anxiety disorder were then grouped into severity-specific sequelae. People with a comorbidity-corrected disability weight of 0 were considered asymptomatic (disability weight 0). Participants with a comorbidity-corrected disability weight greater than 0 and less than the mid-point between the mild and moderate anxiety disorder health state disability weights were considered to have mild anxiety disorder (disability weight 0·030, 95% UI 0·018–0·046). Participants were considered to have severe anxiety disorder (0·523, 0·362–0·677) if their comorbidity-corrected disability weight was greater than the mid-point between the moderate and severe anxiety disorder health state disability weights. All remaining participants were considered to have moderate anxiety disorder (0·133, 0·091–0·186). The proportion of participants sorted to each sequela then represented the severity proportions for anxiety disorders. The sequela-weighted disability weight then effectively represented the overall disability weight for anxiety disorders.

### Grading severity by health-care access

HAQI^10^ was used as a proxy for treatment access for every country in GBD 2019. The HAQI is informed by the mortality rate of 32 causes of death which should not, or should rarely, occur in the presence of effective care. The index spans between 0 (worst) and 100 (best), representing the 1st and 99th percentiles observed since 1990, and every location-year has a corresponding HAQI score. Data on minimally adequate treatment coverage of anxiety disorders from the World Mental Health Surveys were sourced from Alonso and colleagues^14^ and regressed along the HAQI via MR-BRT (appendix p 7). Both linear and logit relationships were explored and the linear relationship best followed the data with the smallest root-mean-square error (appendix pp 9, 14). The HAQI intercept when the treatment coverage was 0% was then considered the maximum HAQI with no population treatment effect (HAQI 44·1, 95% UI 34·2–51·1).

Anxiety disorder comorbidity-corrected disability weights were mapped back to SF-12 and adjusted to reflect likely SF-12 scores if individuals with anxiety disorder had no access to treatment. This was done by multiplying the coverage-adjusted treatment effect size (a weighted SMD) by the SD of the SF-12 scores and adding this to the SF-12 scores, where β represents the coverage-adjusted treatment effect and σ represents the SD of the SF-12 scores.

$$SF12_{no\;treatment,i}=SF12_{i}+\text{β}\times \sigma_{SF\text{12}}$$

SF-12 scores in a no-treatment scenario were then mapped back into disability weights (appendix pp 5–6). The new disability weights then represented the disability weights in the absence of available treatment. Respondents were again grouped into asymptomatic, mild, moderate, and severe categories based on their new disability weights. The proportion of respondents within each sequela and the sequela-weighted disability weight were then calculated.

The proportion of participants with anxiety disorder within each sequela was then linearly interpolated along the HAQI between the proportion estimated in the 1997 NSMHWB (HAQI 88·0, 95% UI 87·5–88·5) and the maximum HAQI with no population treatment effect (44·1, 34·2–51·1), under which proportions were assumed to plateau. Proportions for HAQI scores larger than the HAQI during the 1997 NSMHWB were linearly extrapolated (appendix p 10). Sequela-weighted disability weights were then estimated for every HAQI score that corresponded with every country in GBD 2019.

### Estimating averted and avoidable burden

To estimate the disability weights under a full coverage optimal treatment scenario, the full coverage optimal treatment effect was multiplied by the SD of SF-12 scores, which was then subtracted from the SF-12 scores in the no-treatment scenario

$$SF12_{full\;coverage\;optimal\;treatment,i}=SF12_{no\;treatment,i}+\text{β}\times \sigma_{SF\text{12}}$$

where β represents the full coverage optimal treatment effect and σ represents the SD of the SF-12 scores.

Full coverage optimal treatment SF-12 scores were then mapped back into disability weights to estimate disability weights under a full coverage optimal treatment scenario. These disability weights were again sorted into asymptomatic, mild, moderate, and severe categories and the proportion of participants within each sequela were calculated.

Prevalence estimates for anxiety disorders by location for the year 2019 were sourced from GBD 2019.^1^ Location-specific YLDs were estimated by multiplying the prevalence of anxiety disorders by the sequela-weighted disability weight estimated for the HAQI of that location in 2019. YLDs given a no-treatment scenario were then estimated by multiplying the prevalence of anxiety disorders by the sequela-weighted disability weight for the no-treatment scenario. Averted YLDs were estimated by subtracting the location-specific YLDs from the no-treatment scenario YLDs. Avoidable burden under two scenarios was estimated: 1) best functioning routine health-care services, and 2) full coverage optimal treatment. YLDs under the best functioning routine health-care scenario were estimated by multiplying the prevalence of each disorder by the disability weight estimated for the highest observed HAQI globally in 2019. Avoidable burden under the best functioning routine health-care scenario was then estimated by subtracting the YLDs under a best functioning routine health-care scenario from the location-specific YLDs. YLDs under the full coverage optimal treatment scenario were estimated by multiplying the prevalence of each disorder by the disability weight under the full coverage optimal treatment scenario. Avoidable burden under the full coverage optimal treatment scenario was then estimated by subtracting the YLDs under a full coverage optimal treatment scenario from the location-specific YLDs. Location-specific averted and avoidable YLDs were then population-weighted across countries to provide estimates by super-region and globally.

### Role of the funding source

The funder of the study had no role in study design, data collection, data analysis, data interpretation, or writing of the report.

## Results

**Figure 1:**
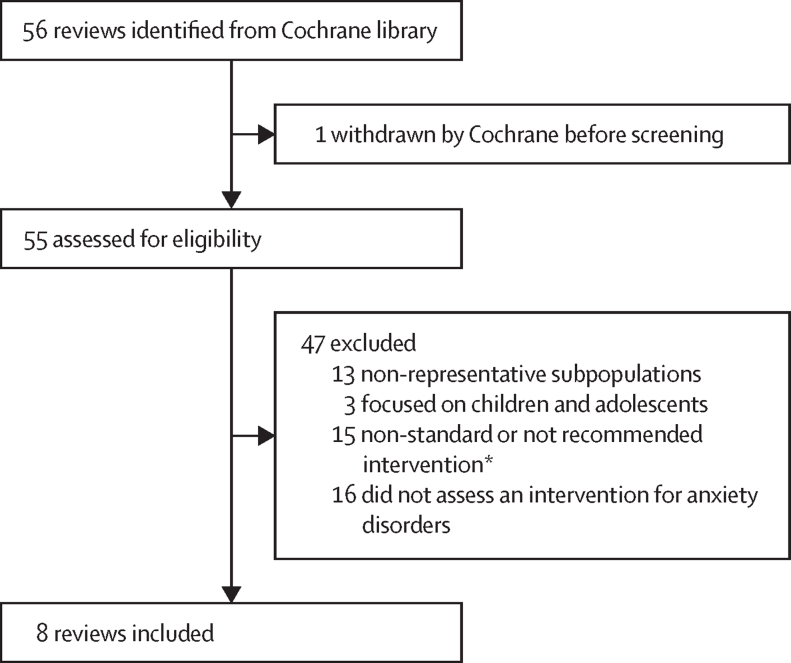
Study selection *Non-standard and not recommended interventions excluded azapirones; antipsychotics; hydroxyzine; d-cycloserine; neurokinins; anticonvulsants; benzodiazepines; valerian; passiflora; therapeutic touch; client feedback during psychological therapy; use of paraprofessionals; routine, patient-reported outcome measures; meditation therapy; repetitive transcranial magnetic stimulation; smoking cessation; and morita therapy.

The Cochrane database search sourced 56 reviews for anxiety disorders, of which eight were eligible for inclusion (figure 1). These eight reviews reported on 156 randomised controlled trials, with 194 unique treatment effects as an SMD on a symptom scale.

**Figure 2:**
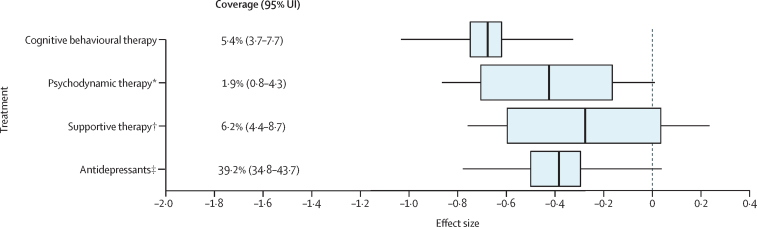
Effect sizes and coverage estimates of treatments for anxiety disorders Boxes represent the 95% UI of the effect size and whiskers represent the 95% UI of the effect size incorporating the unexplained between-study heterogeneity. UI=uncertainty interval. *Coverage of other psychotherapy that was assigned the treatment effect for psychodynamic therapy and consists of patients with anxiety disorder who did not receive cognitive behavioural therapy. †Coverage of counselling that was assigned the treatment effect for supportive therapy and consists of patients with anxiety disorders who did not receive cognitive behavioural therapy or other psychotherapy. ‡Coverage of medication that was assigned the treatment effect for antidepressants.

Among 1997 NSMHWB respondents, the most commonly reported treatment types were medication (39·2%, 95% UI 34·8 to 43·7), followed by supportive therapy (6·2%, 4·4 to 8·7) and CBT (5·4%, 3·7 to 7·8; figure 2). The 1997 Australian survey-weighted treatment effect among participants with anxiety disorder was –0·28 (–0·45 to –0·12). The pooled treatment effect for the full coverage optimal treatment scenario was –1·07 (–1·47 to –0·64). The largest pooled treatment effect for the treatments captured in the NSMHWB was for CBT (*d* –0·68, 95% UI –0·75 to –0·62) followed by psychodynamic therapy (–0·48, –0·81 to –0·21) and antidepressants (–0·38, –0·50 to –0·30; figure 2). The only intervention without a significant treatment effect was supportive therapy (–0·28, –0·60 to 0·03). After incorporating between-study heterogeneity, only the effect size for CBT (–0·68, –1·07 to –0·25) remained significant.

**Figure 3:**
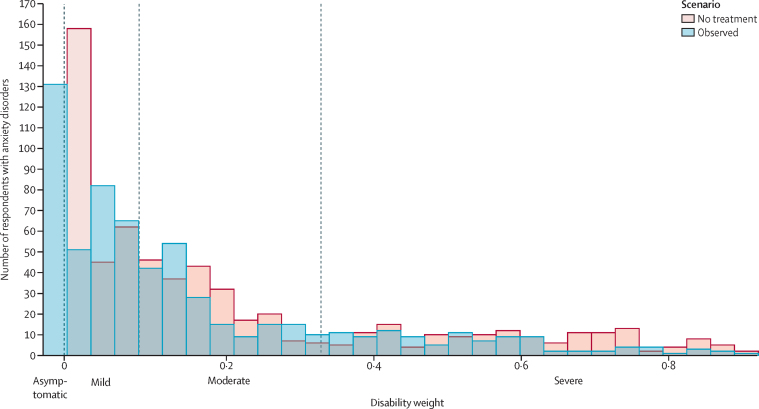
Distribution of disability weights among patients with anxiety disorder in the 1997 Australian National Survey of Mental Health and Wellbeing Dashed lines indicate the cutoff points for the disability weight categories.

The proportion of respondents with anxiety disorder by sequela observed in the 1997 NSMHWB and estimated in the no-treatment and full coverage optimal treatment scenarios is reported in table 1. The sequela-weighted disability weight for responders with anxiety disorder in the 1997 NSMHWB was 0·141 (95% UI 0·042–0·275). After adjusting the severity distribution to reflect the no-treatment scenario (see figure 3 for a visualisation of the distributions), the sequela-weighted disability weight was 0·188 (0·070–0·341), meaning that 26·3% (10·2–45·7) of the non-fatal anxiety disorder burden was averted among patients of the 1997 NSMHWB. The sequela-weighted disability weight if all patients had access to optimal treatment was 0·056 (0·013–0·140). Location-specific sequela-weighted disability weights and severity proportions extrapolated from their respective HAQI scores are presented by GBD super-region in table 2 and by country in the appendix (pp 15–26).

**Table 1:** Estimated severity proportions for anxiety disorders by scenario

	**Disability weight**	**Observed in 1997 NSMHWB (%)**	**No treatment (%)**	**Full coverage optimal treatment (%)**
Asymptomatic	0·000 (0·000–0·000)	23·0% (4·0–55·2)	0·0% (0·0–0·0)	34·5% (7·4–70·2)
Mild	0·030 (0·018–0·046)	29·5% (16·1–39.5)	43·7 % (14·8–80·7)	40·3% (18·5–64·5)
Moderate	0·133 (0·091–0·186)	29·3% (12·3–43·7)	30·0% (12·6–43·6)	20·5% (3·1–46·0)
Severe	0·523 (0·362–0·677)	18.2% (3·1–45·0)	26·3% (5·6–59·2)	3·5% (0·0–17·8)

Data are n (95% UI) or % (95% UI). UI=uncertainty interval. NSMHWB=National Survey of Mental Health and Wellbeing.

**Table 2:** Change in disability weights and severity proportions for anxiety disorders for 2019 by super-region and globally

	Adjusted disability weight*	**Change in disability weight (%)**	**Severity (%)**			
			Asymptomatic	Mild	Moderate	Severe
Global	0·166 (0·056 to 0·309)	20·0 (6·0 to 45·0)	9·6 (1·7 to 22·8)	37·8 (15·6 to 59·8)	29·7 (12·6 to 42·6)	22·9 (4·5 to 53·0)
Central Europe, eastern Europe, and central Asia	0·155 (0·051 to 0·294)	11·8 (3·6 to 27·6)	15·2 (2·6 to 36·2)	34·3 (15·6 to 49·5)	29·5 (12·9 to 42·6)	21·0 (3·9 to 48·4)
High income	0·139 (0·041 to 0·272)	−1·8 (−4·3 to −0·5)	24·1 (4·3 to 58·0)	28·8 (16·0 to 38·6)	29·2 (12·2 to 44·0)	17·8 (2·9 to 44·3)
Latin America and Caribbean	0·169 (0·060 to 0·316)	23·2 (6·9 to 53·1)	9·1 (1·5 to 21·9)	38·1 (15·4 to 61·2)	29·7 (12·6 to 42·5)	23·1 (4·6 to 53·3)
North Africa and Middle East	0·167 (0·058 to 0·314)	21·6 (6·3 to 50·1)	9·6 (1·6 to 23·1)	37·8 (15·5 to 60·3)	29·7 (12·6 to 42·6)	22·9 (4·5 to 52·8)
South Asia	0·187 (0·070 to 0·341)	37·7 (11·2 to 83·7)	0·3 (0·0 to 2·7)	43·5 (14·8 to 79·7)	30·0 (12·6 to 43·5)	26·1 (5·6 to 58·7)
Southeast Asia, east Asia, and Oceania	0·160 (0·053 to 0·302)	15·6 (4·7 to 35·6)	14·0 (2·4 to 33·1)	35·1 (15·6 to 51·4)	29·5 (12·8 to 42·5)	21·4 (4·0 to 49·4)
Sub-Saharan Africa	0·188 (0·070 to 0·341)	37·9 (11·3 to 84·3)	0·1 (0·0 to 0·6)	43·7 (14·8 to 80·4)	30·0 (12·6 to 43·5)	26·2 (5·6 to 59·2)

Data are n (95% UI) or % (95% UI). UI=uncertainty interval.

* Original disability weight 0·141 (0·042 to 0·275).

**Figure 4:**
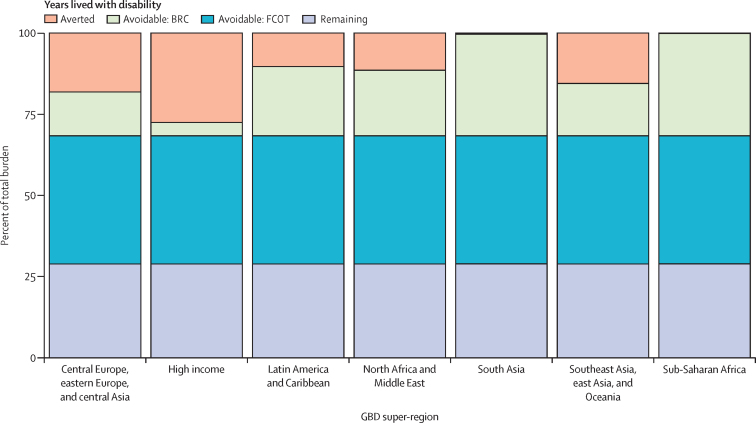
Proportion of anxiety disorder burden averted, avoidable, and remaining in 2019 by GBD super-region BRC=best routine care scenario. FCOT=full coverage optimal treatment scenario. GBD=Global Burden of Disease Study.

Globally, 12·5% of anxiety disorder burden (95% UI 4·6–21·5) was estimated to have been averted due to available treatment. The high-income GBD super-region had the greatest proportion of burden of anxiety disorder that was averted in 2019 due to treatment (27·5%, 10·7–48·3), followed by central Europe, eastern Europe, and central Asia (18·2%, 6·8–31·3), and Southeast Asia, east Asia, and Oceania (15·5%, 5·7–26·5; figure 4). The GBD super-regions with the lowest averted burden were sub-Saharan Africa (0·2%, 0·0–0·7) and south Asia (0·4%, 0·0–3·1). Latin America and the Caribbean averted 10·3% (3·6–18·7) of anxiety disorder burden and North Africa and the Middle East averted 11·5% (4·1–20·5). Globally, 31·7% of anxiety disorder burden (12·3–55·7) was avoidable in the best functioning routine health care scenario. However, 71·1% of global burden (46·2–87·6) could be averted if all people with anxiety disorder had access to optimal treatment (figure 4).

## Discussion

In this paper we demonstrated a novel method that builds upon GBD methods to estimate location-specific disorder severity using anxiety disorders as a case study, and showed its extended applications for estimating averted and avoidable burden. Among the treatment effects captured within the NSMHWB, we estimated CBT to be the most effective psychotherapy for anxiety disorders. Psychodynamic therapy (assigned to other psychotherapy) was the second largest, followed by antidepressants.

We estimated more than 25% of anxiety disorder burden was averted in the NSMHWB, double previous estimates by Andrews and colleagues that used the same survey.^12^ There are several differences in methods that probably explain this difference. First, Andrews and colleagues only considered respondents who reported their anxiety disorder as their primary complaint. However, we included all participants with anxiety disorder (regardless of primary complaint) and estimated the disability specific to anxiety disorders for patients with comorbid conditions. Andrews and colleagues also considered fewer treatment options as effective for anxiety disorders (eg, only considering CBT and not supportive or other psychological therapy).

Anxiety disorder severity and burden were estimated to be lowest in high-income countries, which have higher health-care access quality. People with anxiety disorder in sub-Saharan Africa and south Asia were estimated to experience the highest severity and burden. However, despite effective treatments available, low treatment uptake among people with anxiety disorder meant the coverage-adjusted treatment effect was small. Even in the best functioning routine health care scenario, low treatment use in people with anxiety disorder meant a substantial proportion of burden was still avoidable. More than double the burden avoidable in the best functioning routine health care scenario could be averted in the full coverage optimal treatment scenario.

The substantial burden avoidable in the full coverage optimal treatment scenario highlights the importance of public promotion and referral pathways for available treatments for anxiety disorders. Anxiety disorders were among the leading causes of non-fatal burden globally in 2019,^1^ and the emergence of COVID-19 in 2020 is estimated to have increased the global burden of anxiety disorders by 25·5%.^24^ Despite evidence that effective prevention and interventions exist, most mental health systems globally are under-resourced, disorganised, and geared towards treatment of more severe mental disorders in specialised health-care settings.^9^, ^11^, ^25^, ^26^ The COVID-19 pandemic probably exacerbated this issue, as inpatient and outpatient services were interrupted or redirected to treat cases of COVID-19.^27^, ^28^, ^29^, ^30^ Even public health strategies to mitigate virus spread have probably caused difficulties in receiving treatments such as medications or in-person care. Improving treatment coverage for anxiety disorders should be a public health priority.

Our study had several limitations. First, anxiety disorder prevalence and burden within GBD 2019 were modelled for a single cause of any anxiety disorder and so we did this analysis pooling treatment effects across all anxiety disorder diagnoses. However, anxiety disorders consist of several heterogeneous diagnoses varying in characteristics, symptom triggers, and responsiveness to treatments. As this was a proof-of-concept work, and to align with the GBD case definitions, we took a simplistic approach to do this analysis across all anxiety disorders. We also did not explore how treatment effects, service utilisation, or averted or avoidable burden might vary by sex. Next steps could include refinement of the estimates to reflect specific anxiety disorder diagnoses by sex.

Second, we estimated treatment effects as SMDs on anxiety symptom scales and assumed these to be generalisable to SF-12 scores. This is the accepted assumption behind pooling SMDs in a meta-analysis.^31^ However, in practice the generalisability of the SMD between two scales is dependent on the correlation between them. Few studies have reported moderate correlations between SF-12 scores and anxiety symptom scales.^32^, ^33^ Third, the HAQI is estimated from death rates of preventable causes of mortality.^10^ Although it is the best available proxy for health-care access quality for every location within GBD 2019, there will still be heterogeneity in mental health service availability across countries with similar HAQI scores, depending on the country-specific policy and funding priorities. Fourth, this work relied on the assumption that treatment access is the dominant source of geographical variation in mental disorder severity. Although modelling exercises suggest treatment access improves population mental health,^7^ this is yet to be observed with real-world data and might be due to insufficient coverage of minimally adequate treatment.^9^ Fifth, this work assumed that the treatment effects sourced from randomised controlled trials were applicable at the population level. However, we acknowledge that efficacy of an intervention does not necessarily equal its effectiveness in the real world. Real-world application of interventions can vary substantially from randomised controlled trials in dosing and treatment environment, and people with anxiety disorder in trials might not be representative of the broader population. Sixth, the 12-month recall period for reporting service use in the 1997 NSMHWB did not align with the 30-day recall period for people with anxiety disorder used to align with the GBD case definition and estimates. This might have overestimated the treatment effect estimated for some respondents with anxiety disorder in the survey.

Despite these limitations, our method is useful for both simulating location-specific severity distributions for GBD and simulating averted and avoidable burden globally. GBD provides a snapshot for policy makers, service planners, resource allocators, and researchers globally to rank causes of burden to prioritise. The addition of location-specific severity distributions for anxiety disorders in GBD greatly increases precision in burden estimates for non-high-income countries and addresses a key recommendation for methods development for mental disorders in GBD.^26^ Further work is warranted to apply this method to other causes of burden in GBD. Additionally, GBD does not provide information to indicate which causes of burden should be prioritised based on avoidable burden. High but untreatable causes of burden could garner more attention than lower but treatable causes of burden. By deconstructing non-fatal burden into components that have been averted with current systems, those that could be avoided with additional treatment coverage and those that cannot be avoided using existing treatments, this work provides a roadmap for clinicians, public health practitioners, and policy makers to translate GBD findings into actionable results.

## Data sharing

To download code and data used in these analyses, please visit the GitHub page (https://github.com/ihmeuw/mental_disorders/tree/anxiety_disorders_severity_by_treatment_access). Prevalence and YLDs of anxiety disorders from GBD 2019 are available for download via the GBD Results Tool (https://vizhub.healthdata.org/gbd-results/). Access to the 1997 NSMHWB is available by application to the Australian Bureau of Statistics.

## Declaration of interests

We declare no competing interests.
