# Supplementary material for: Grading disorder severity and averted burden by access to treatment within the GBD framework: a case study with anxiety disorders
Source: Lancet Psychiatry. 2023 Apr;10(4):272–81. doi: 10.1016/S2215-0366(23)00037-8 (PMC10017349; doi:10.1016/S2215-0366(23)00037-8)
Supplement: Supplementary appendix [file mmc1.pdf]

THE LANCET
Psychiatry

Supplementary appendix

This appendix formed part of the original submission and has been peer reviewed. We post it as supplied by the authors.

Supplement to: Santomauro DF, Purcell C, Whiteford HA, Ferrari AJ, Vos T. Grading disorder severity and averted burden by access to treatment within the GBD framework: a case study with anxiety disorders. *Lancet Psychiatry* 2023; **10:** 272–81.

Contents

List of figures and tables 2
Figures 2
Tables 2
Section 1. Evidence for cumulative treatment effects 3
Section 2. Assigning optimal treatment effect size to all anxiety disorder cases 4
Section 3. Mapping 12-item Short Form Health Survey to disability weights 5
Overview 5
New method 6
Section 4. Relationship between the healthcare access quality index and treatment coverage of anxiety disorders 7
Section 5. Figures and tables 8
Figure S1: Overview of the methodology to estimate anxiety disorder disability weights, severity, and years lived with disability 8
Figure S2: Meta-regressions of HAQI scores on MAT coverage for anxiety disorders in MR-BRT 9
Figure S3: Proportion of anxiety disorder cases in each sequela weight by healthcare access quality index 10
Figure S4: Regression of individual-level SF-12 scores on disability weights via quadratic spline in MR-BRT 10
Figure S5: Regression of aggregate-level SF-12 scores on disability weights via quadratic spline in MR-BRT 10
Figure S6: Regression of individual-level SF-12 scores on disability weights via quadratic spline in MR-BRT using spline priors from aggregate model 11
Table S1: Guidelines for Accurate and Transparent Health Estimates Reporting (GATHER) checklist 12
Table S2: Results of the meta-regression of MAT and HAQI 14
Table S3: Change in disability weights for 2019 by country, region, and super-region 15
Table S4: Severity proportions for anxiety disorders for 2019 by country, region, and super-region 21

List of figures and tables

Figures

Figure S1: Overview of the methodology to estimate anxiety disorder disability weights, severity, and years lived with disability

Figure S2: Meta-regressions of HAQI scores on MAT coverage for anxiety disorders in MR-BRT

Figure S3: Proportion of anxiety disorder cases in each sequela weight by healthcare access quality index

Figure S4: Regression of individual-level SF-12 scores on disability weights via quadratic spline in MR-BRT

Figure S5: Regression of aggregate-level SF-12 scores on disability weights via quadratic spline in MR-BRT

Figure S6: Regression of individual-level SF-12 scores on disability weights via quadratic spline in MR-BRT using spline priors from aggregate model

Tables

Table S1: Guidelines for Accurate and Transparent Health Estimates Reporting (GATHER) checklist

Table S2: Results of the meta-regression of MAT and HAQI

Table S3: Change in disability weights for 2019 by country, region, and super-region

Table S4: Severity proportions for anxiety disorders for 2019 by country, region, and super-region

Section 1. Evidence for cumulative treatment effects

We inspected the treatment effects of combined treatments reported by the Cochrane reviews of randomized controlled trials (RCTs) for anxiety disorders to determine whether it would be appropriate to assume treatment effects of medication and psychotherapy were additive. Hetrick and colleagues estimated a pooled effect size from two RCTs for cognitive behavioural therapy (CBT) and antidepressants together vs antidepressants alone of -0.39 [95% Uncertainty Interval: -0.85 to 0.07].[1] Additionally, one RCT reported in Abbas and colleagues reported the combined treatment effect for both psychodynamic therapy and antidepressants vs antidepressants alone and reported a large additional treatment effect -1.32 (-2.01 to -0.63).[3]

Together these estimates suggested it would be appropriate to assume the effect size of psychotherapy and medication would at least be greater than the effect size of the most effective treatment. To what extent these effects would be additive was difficult to determine. Thorlund and Mills concluded from their simulation study investigating the assumption of additive treatment effects that assuming treatment effects are additive is reasonable if the assumption of approximate additivity is sensible.[5] Additionally Mills and colleagues concluded it is appropriate and useful to assume treatment effects are additive as long as the treatments do not overlap or interact in their treatment mechanisms or biological pathways.[6] For this reason we did not assume that effect sizes between psychotherapies were additive. Instead, assumed a hierarchy whereby supportive therapy was only assigned in the absence of CBT and psychodynamic therapy and psychodynamic therapy was only assigned in the absence of CBT. Overall these effect sizes did not seem substantially different from the pooled treatment effect sizes from the current study, and so we considered it reasonable for the current paper to assume treatment effects would be additive.

Section 2. Assigning optimal treatment effect size to all anxiety disorder cases

Every anxiety disorder case in the *full coverage optimal treatment* scenario was assigned the cumulative treatment effect for CBT and antidepressants. However, we acknowledge that in practice not every anxiety disorder case should be provided both antidepressants and CBT in combination. Disability weights of cases are floored at 0, and there would be cases within the 1997 NSMHWB that would reach a disability weight of 0 with either antidepressants or CBT alone (not both). Because disability weights are floored at 0, assigning these cases the *optimal treatment* effect size would reach the same disability weight as CBT or antidepressants alone (depending on which would work best for the case). We can therefore assume within our simulation of the *full coverage optimal treatment* scenario that cases that reach a disability weight of 0 with either CBT or antidepressants alone would only receive the treatment necessary for their level of disability.

Section 3. Mapping 12-item Short Form Health Survey to disability weights

Overview

For GBD 2013, a convenience sample was asked to complete completed the 12-item Short Form Health Survey (SF-12)[7] forms for hypothetical individuals experiencing specific health states. Each respondent completed SF-12 forms for the lay descriptions of up to 50 randomly selected health states (e.g., "feels anxious and worried, which makes it difficult to concentrate, remember things, and sleep. The person tires easily and finds it difficult to perform daily activities"). The health states were randomly selected from a selection of 62 health states that represented the entire severity spectrum of disability weight used in GBD. These SF-12 scores were then regressed on their respective disability weights to create a model to predict disability weights by SF-12 scores. The analytical method used in the current paper to map SF-12 scores to disability weights from this data differed from the analytical method historically used for GBD 2013, 2015, 2016, 2017, and 2019.

Original analytical method

There was substantial heterogeneity among the SF-12 scores and so SF-12 scores more than 2 median absolute deviations from the median for each health state were excluded (19% of observations). The average SF-12 score for each health state was then calculated from the remaining SF-12 observations and a loess regression was conducted to regress the average SF-12 scores on their respective disability weights.

The above approach had several limitations that we wished to address for the current paper. First, the exclusion of SF-12 scores based on the median absolute deviation criteria led to the exclusion of a large proportion of observations. Second, disability weights were not logit transformed prior to analysis, meaning SF-12 scores could be mapped to a disability weight below 0 or above 1. This meant disability weights below 0 or above 1 had to be manually capped at 0 and 1 respectively. Third, the loess regression did not incorporate the uncertainty in the disability weights. Last, the regression was conducted on the average SF-12 scores rather than the individual response data and so did not incorporate the uncertainty in the individual responses.

New method

To correct for the above limitations, we regressed SF-12 scores on the logit-transformed disability weights via a quadratic spline using Meta Regression: Bayesian, Regularized, Trimmed[8] (MR-BRT). A quadratic spline was used to capture the non-linear relationship between SF-12 scores and disability weights. Several steps were taken to achieve a model that followed the health state disability weights. First, for each health state, SF-12 scores ±1.64 standard deviations from the mean (representing the top and bottom 5% of the estimates) were excluded (9% of observations). A model using individual-level SF-12 scores was tested but the spline failed to reach health states with a disability weight above 0.5 due to the substantial number of observations for health states with a disability weight below 0.5 (Figure S4). We then calculated the average SF-12 score for each health state and conducted a model with this aggregate data. This provided a fit that followed the disability weights well across the entire spectrum (Figure S5). However, the aggregation of the SF-12 scores by health state meant this model ignored the heterogeneity of the individual observations. To accommodate for this, we re-ran the model with individual-level SF-12 scores and used the intercept and spline coefficients from the aggregate model as priors, and included gamma (between-observation heterogeneity) in the prediction uncertainty. This resulted in a final model that followed disability weights well across the entire spectrum with appropriate uncertainty (Figure S6). Finally, we assumed an SF-12 score of 120 (the maximum observed score in the 1997 NSMHWB) equated a disability weight of 0.

Section 4. Relationship between the healthcare access quality index and treatment coverage of anxiety disorders

Estimates of minimally adequate treatment (MAT) coverage from the World Mental Health Surveys were sourced from Alonso and colleagues (2018)[9] and the HAQI corresponding to the location-year of each survey were sourced from GBD 2019. There were 23 estimates of MAT across 21 countries. We regressed Healthcare Access Quality Index (HAQI) scores on MAT coverage via Meta Regression: Bayesian, Regularized, Trimmed[8] (MR-BRT). The estimate for Nigeria was 0% with no measure of uncertainty and so could not be included in the meta-regression. Both linear and logit relationships were explored and both indicated a significant relationship between the HAQI and MAT coverage (Table S2). However the linear relationship had the smallest root-mean-square error and visual inspection of both model fits supported the model fit of the linear model suggesting there should be a non-zero HAQI score at which we should assume 0% for effective treatment (Figure S2). We pulled 1000 samples of the HAQI intercept when treatment coverage was 0 (HAQI = 44·1 [95% UI: 34·2–51·1]) and this was considered the maximum HAQI with no population treatment effect.

Section 5. Figures and tables

Figure S1: Overview of the methodology to estimate anxiety disorder disability weights, severity, and years lived with disability

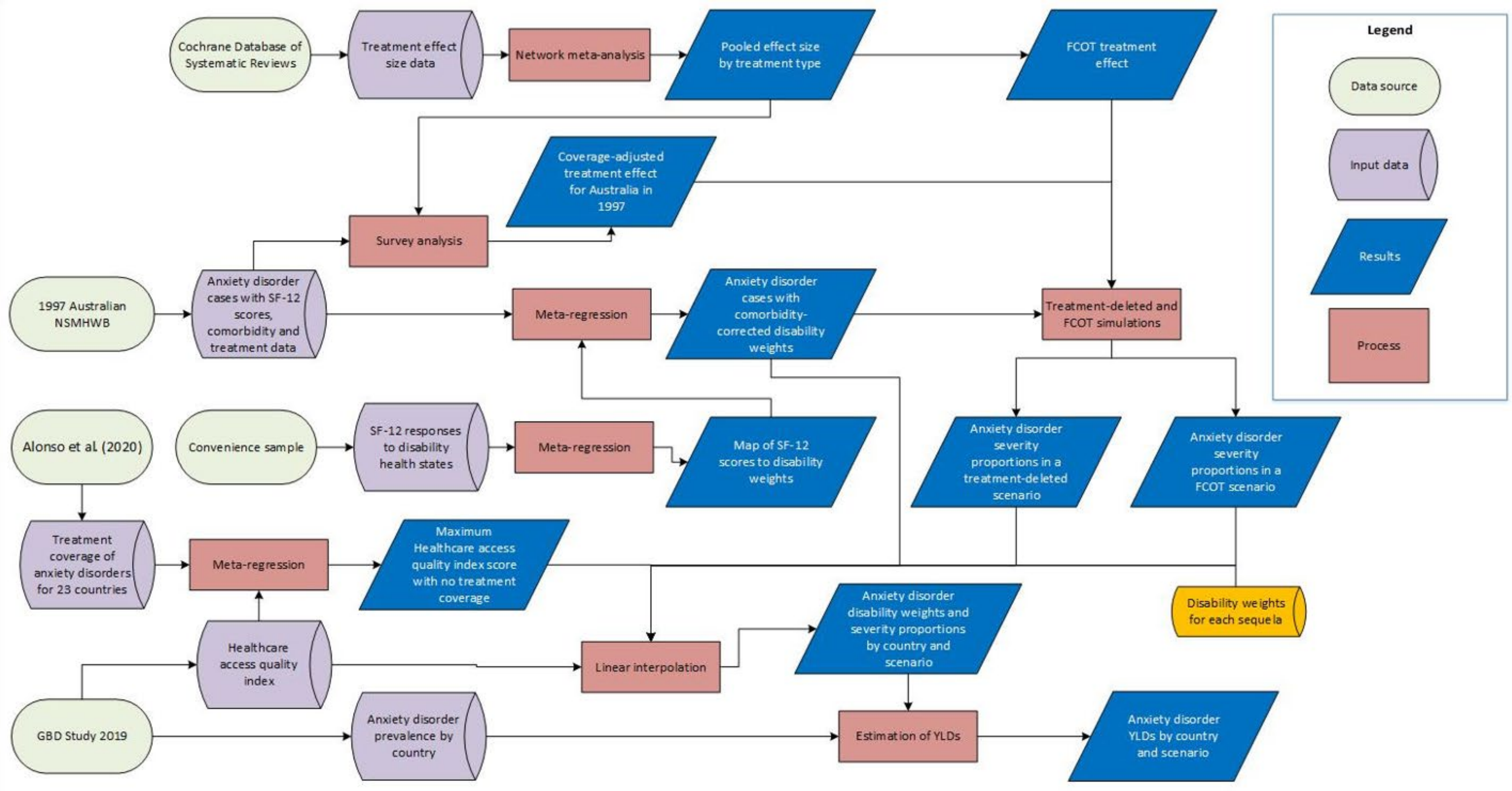

GBD = Global Burden of Disease. FCOT = Full coverage optimal treatment. NSMHWB = National Survey of Mental Health and Wellbeing. YLD = Years lived with disability.

Figure S2: Meta-regressions of HAQI scores on MAT coverage for anxiety disorders in MR-BRT

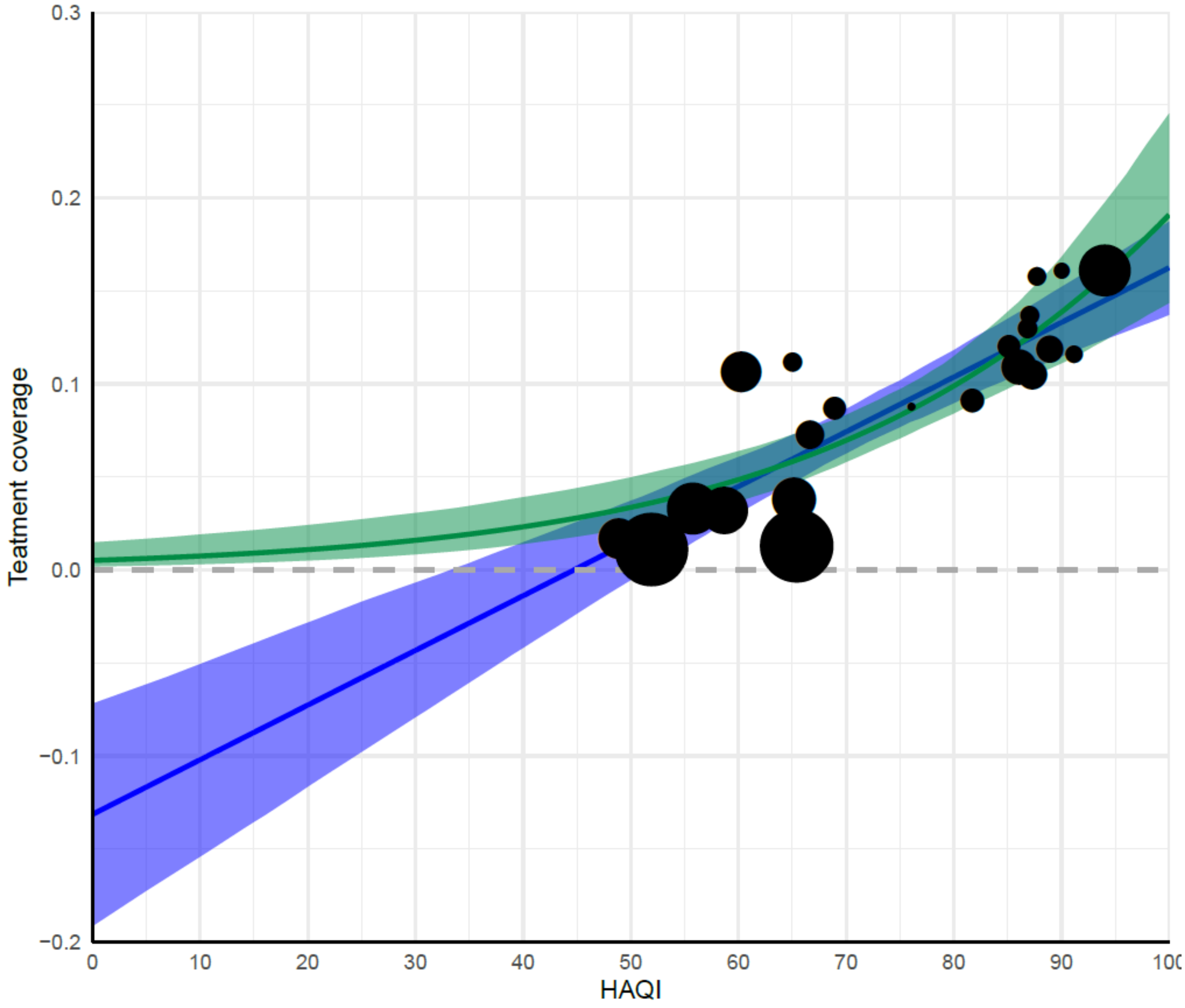

Black points = Study estimates. Blue line = Predictions from linear model. Blue ribbons = 95% uncertainty interval from linear model. Green line = Predictions from logit model. Green ribbons = 95% uncertainty interval from logit model. HAQI = Healthcare Access Quality Index. MAT = Minimally adequate treatment.

Figure S3: Proportion of anxiety disorder cases in each sequela weight by healthcare access quality index

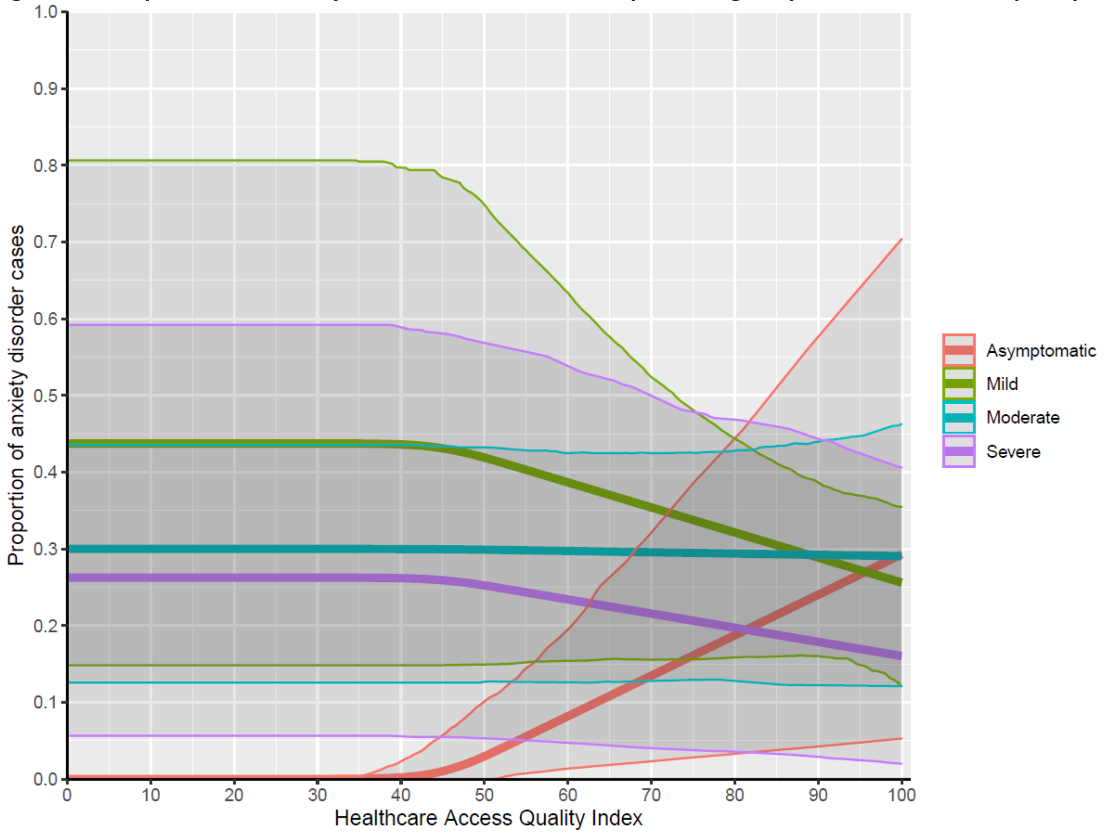

Figure S4: Regression of individual-level SF-12 scores on disability weights via quadratic spline in MR-BRT

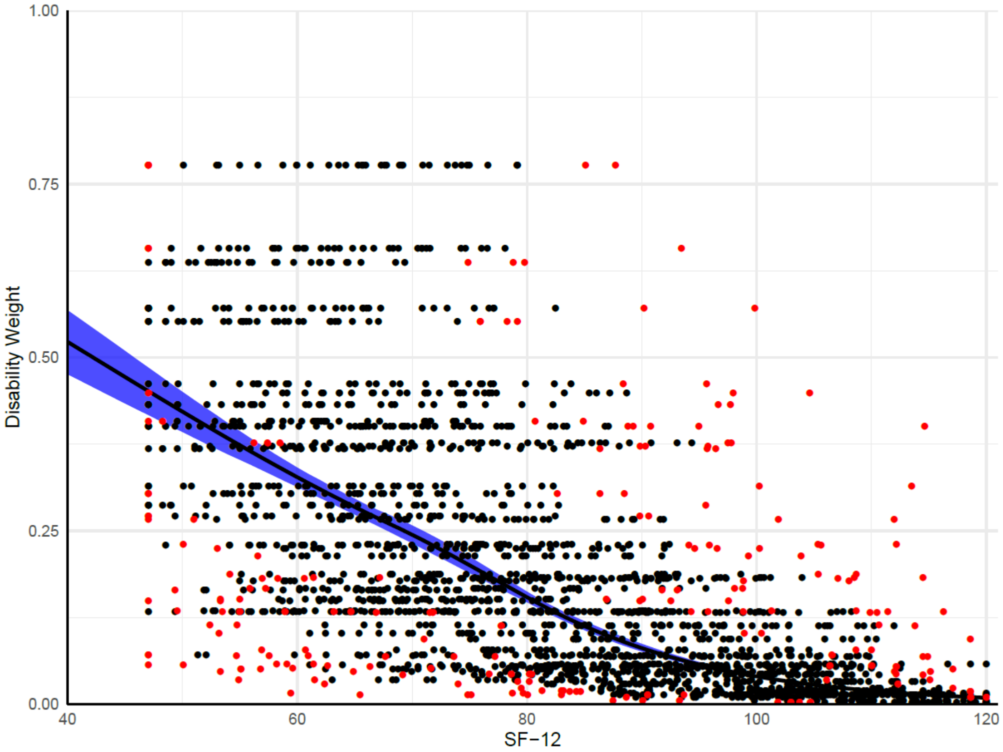

Black points = SF-12 scores. Red points = Outliered SF-12 scores. SF-12 = 12-item Short Form Health Survey.

Figure S5: Regression of aggregate-level SF-12 scores on disability weights via quadratic spline in MR-BRT

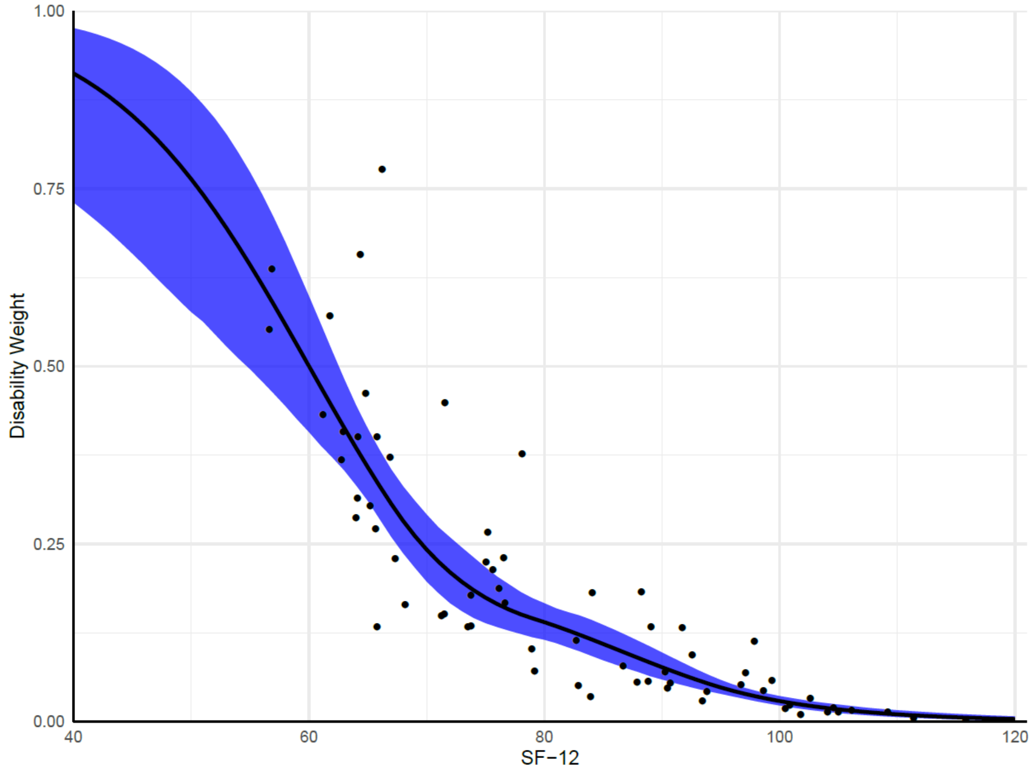

Black points = Average SF-12 scores by health state. SF-12 = 12-item Short Form Health Survey.

Figure S6: Regression of individual-level SF-12 scores on disability weights via quadratic spline in MR-BRT using spline priors from aggregate model

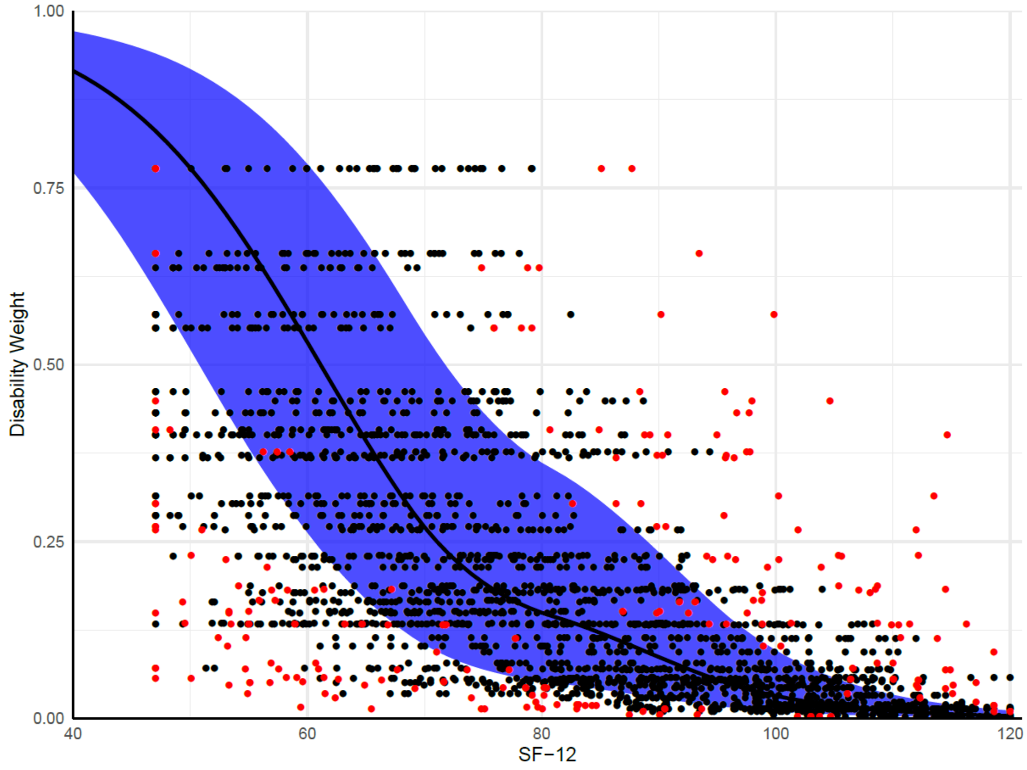

Table S1: Guidelines for Accurate and Transparent Health Estimates Reporting (GATHER) checklist

Item #	Checklist item	Location
**Objectives and funding**		
1	Define the indicator(s), populations (including age, sex, and geographic entities), and time period(s) for which estimates were made.	Background p 2-3<br>Method p 3
2	List the funding sources for the work.	Acknowledgements p 9
**Data Inputs**		
For all data inputs from multiple sources that are synthesized as part of the study:		
3	Describe how the data were identified and how the data were accessed.	Method pp 3-4
4	Specify the inclusion and exclusion criteria. Identify all ad-hoc exclusions.	Method p 3
5	Provide information on all included data sources and their main characteristics. For each data source used, report reference information or contact name/institution, population represented, data collection method, year(s) of data collection, sex and age range, diagnostic criteria or measurement method, and sample size, as relevant.	https://github.com/ihmeuw/mental_disorders/tree/anxiety_disorders_severity_by_treatment_access
6	Identify and describe any categories of input data that have potentially important biases (e.g., based on characteristics listed in item 5).	Method p 3-4
For data inputs that contribute to the analysis but were not synthesized as part of the study:		
7	Describe and give sources for any other data inputs.	Method pp 3-5
For all data inputs:		
8	Provide all data inputs in a file format from which data can be efficiently extracted (e.g., a spreadsheet rather than a PDF), including all relevant meta-data listed in item 5. For any data inputs that cannot be shared because of ethical or legal reasons, such as third-party ownership, provide a contact name or the name of the institution that retains the right to the data.	https://github.com/ihmeuw/mental_disorders/tree/anxiety_disorders_severity_by_treatment_access
**Data analysis**		
9	Provide a conceptual overview of the data analysis method. A diagram may be helpful.	Method pp 3<br>Figure S1
10	Provide a detailed description of all steps of the analysis, including mathematical formulae. This description should cover, as relevant, data cleaning, data pre-processing, data adjustments and weighting of data sources, and mathematical or statistical model(s).	Method pp 3-6<br>Appendix pp 5-7

11	Describe how candidate models were evaluated and how the final model(s) were selected.	Method pp 3, 5<br>Appendix pp 5-7
12	Provide the results of an evaluation of model performance, if done, as well as the results of any relevant sensitivity analysis.	Appendix p 7
13	Describe methods for calculating uncertainty of the estimates. State which sources of uncertainty were, and were not, accounted for in the uncertainty analysis.	Method p 3
14	State how analytic or statistical source code used to generate estimates can be accessed.	https://github.com/ihmeuw/mental_disorders/tree/anxiety_disorders_severity_by_treatment_access
**Results and Discussion**		
15	Provide published estimates in a file format from which data can be efficiently extracted.	Table S3<br>Table S4<br>https://github.com/ihmeuw/mental_disorders/tree/anxiety_disorders_severity_by_treatment_access
16	Report a quantitative measure of the uncertainty of the estimates (e.g. uncertainty intervals).	*All estimates are provided with 95% uncertainty intervals.*
17	Interpret results in light of existing evidence. If updating a previous set of estimates, describe the reasons for changes in estimates.	Discussion pp 8
18	Discuss limitations of the estimates. Include a discussion of any modelling assumptions or data limitations that affect interpretation of the estimates.	Discussion p 8-9

Table S2: Results of the meta-regression of MAT and HAQI

Relationship	RMSE	Covariate	Coefficient	95% *UI*	*p*
Linear	0.025	Intercept	-0.131	-0.190 to -0.073	< 0.0001
		HAQI	0.003	0.002 to 0.004	< 0.0001
Logit	0.432	Intercept	-5.271	-6.310 to -4.232	< 0.0001
		HAQI	0.038	0.025 to 0.051	< 0.0001

HAQI = Healthcare access quality index. MAT = Minimally adequate treatment. RMSE = Root-mean-square error. UI = Uncertainty interval.

Table S3: Change in disability weights for 2019 by country, region, and super-region

Location	HAQI	Original DW	Adjusted DW	DW % change
**Global**	**59·7 (59·3–60·1)**	**0·141 (0·042–0·275)**	**0·166 (0·056–0·309)**	**20·0 (6·0–45·0)**
**Central Europe, eastern Europe, and central Asia**	**73·2 (72·6–73·8)**	**0·141 (0·042–0·275)**	**0·155 (0·051–0·294)**	**11·8 (3·6–27·6)**
**Central Asia**	**60·1 (59·5–60·9)**	**0·141 (0·042–0·275)**	**0·171 (0·060–0·318)**	**24·3 (7·3–56·7)**
Armenia	69·8 (68·5–71·5)	0·141 (0·042–0·275)	0·161 (0·054–0·303)	16·0 (4·8–38·1)
Azerbaijan	59·8 (58·0–61·0)	0·141 (0·042–0·275)	0·171 (0·061–0·319)	24·8 (7·3–57·6)
Georgia	63·8 (62·0–65·5)	0·141 (0·042–0·275)	0·167 (0·058–0·314)	21·2 (6·4–49·1)
Kazakhstan	67·5 (66·0–69·0)	0·141 (0·042–0·275)	0·163 (0·055–0·307)	18·0 (5·3–41·5)
Kyrgyzstan	59·8 (58·5–61·5)	0·141 (0·042–0·275)	0·171 (0·061–0·318)	24·8 (7·4–56·3)
Mongolia	51·7 (50·0–53·5)	0·141 (0·042–0·275)	0·180 (0·066–0·332)	31·9 (9·3–74·3)
Tajikistan	48·2 (46·5–50·0)	0·141 (0·042–0·275)	0·184 (0·068–0·336)	34·6 (10·3–80·4)
Turkmenistan	58·7 (57·0–60·5)	0·141 (0·042–0·275)	0·173 (0·061–0·320)	25·7 (7·7–60·2)
Uzbekistan	59·5 (58·0–61·0)	0·141 (0·042–0·275)	0·172 (0·060–0·321)	25·1 (7·5–58·5)
**Central Europe**	**80·5 (80·1–81·0)**	**0·141 (0·042–0·275)**	**0·149 (0·047–0·282)**	**6·6 (2·0–15·0)**
Albania	78·0 (77·0–79·5)	0·141 (0·042–0·275)	0·152 (0·048–0·287)	8·8 (2·7–19·7)
Bosnia and Herzegovina	74·4 (73·0–75·5)	0·141 (0·042–0·275)	0·156 (0·051–0·292)	11·9 (3·5–27·7)
Bulgaria	73·8 (72·5–75·0)	0·141 (0·042–0·275)	0·156 (0·051–0·295)	12·5 (3·7–28·3)
Croatia	88·4 (87·5–89·5)	0·141 (0·042–0·275)	0·141 (0·041–0·275)	-0·3 (-1·8–0·7)
Czech Republic	87·6 (86·5–88·5)	0·141 (0·042–0·275)	0·141 (0·042–0·275)	0·4 (-0·6–1·7)
Hungary	82·4 (81·5–83·5)	0·141 (0·042–0·275)	0·147 (0·045–0·281)	4·9 (1·5–11·1)
Montenegro	81·1 (80·0–82·0)	0·141 (0·042–0·275)	0·148 (0·046–0·282)	6·1 (1·8–13·9)
North Macedonia	75·4 (74·0–76·5)	0·141 (0·042–0·275)	0·155 (0·050–0·291)	11·0 (3·3–25·7)
Poland	80·9 (80·0–82·0)	0·141 (0·042–0·275)	0·149 (0·046–0·282)	6·3 (1·8–14·1)
Romania	76·8 (75·5–78·0)	0·141 (0·042–0·275)	0·153 (0·049–0·290)	9·9 (3·0–23·0)
Serbia	78·5 (77·5–79·5)	0·141 (0·042–0·275)	0·151 (0·048–0·286)	8·4 (2·5–19·3)
Slovakia	82·8 (81·5–84·0)	0·141 (0·042–0·275)	0·147 (0·045–0·281)	4·6 (1·3–10·6)
Slovenia	91·6 (91·0–92·5)	0·141 (0·042–0·275)	0·137 (0·039–0·268)	-3·2 (-7·5–-0·9)
**Eastern Europe**	**75·1 (74·0–76·1)**	**0·141 (0·042–0·275)**	**0·155 (0·050–0·292)**	**11·4 (3·4–26·2)**
Belarus	80·1 (78·5–81·5)	0·141 (0·042–0·275)	0·149 (0·047–0·283)	6·9 (2·1–15·7)
Estonia	84·1 (82·5–85·5)	0·141 (0·042–0·275)	0·145 (0·044–0·279)	3·4 (1·0–7·8)
Latvia	77·2 (76·0–79·0)	0·141 (0·042–0·275)	0·153 (0·049–0·288)	9·4 (2·7–21·3)
Lithuania	75·8 (74·0–77·5)	0·141 (0·042–0·275)	0·154 (0·050–0·291)	10·7 (3·2–24·4)
Moldova	67·9 (66·0–69·5)	0·141 (0·042–0·275)	0·163 (0·056–0·306)	17·7 (5·3–40·9)
Russia	75·5 (74·0–77·0)	0·141 (0·042–0·275)	0·154 (0·050–0·292)	11·0 (3·3–25·5)
Ukraine	72·8 (71·5–74·5)	0·141 (0·042–0·275)	0·157 (0·052–0·297)	13·4 (4·0–30·2)
**High income**	**90·2 (89·8–90·6)**	**0·141 (0·042–0·275)**	**0·139 (0·041–0·272)**	**-1·8 (-4·3–-0·5)**
**Australasia**	**94·6 (94·0–95·0)**	**0·141 (0·042–0·275)**	**0·134 (0·038–0·263)**	**-5·7 (-13·5–-1·7)**
Australia	95·1 (94·5–95·5)	0·141 (0·042–0·275)	0·133 (0·037–0·262)	-6·2 (-14·6–-1·8)
New Zealand	91·8 (91·0–92·5)	0·141 (0·042–0·275)	0·137 (0·039–0·269)	-3·3 (-7·8–-0·9)
**High-income Asia Pacific**	**93·0 (92·4–93·5)**	**0·141 (0·042–0·275)**	**0·136 (0·039–0·268)**	**-4·2 (-9·8–-1·2)**
Brunei	68·3 (67·0–69·5)	0·141 (0·042–0·275)	0·162 (0·055–0·306)	17·3 (5·2–40·7)
Japan	93·9 (93·0–94·5)	0·141 (0·042–0·275)	0·135 (0·038–0·265)	-5·2 (-11·9–-1·5)
South Korea	91·3 (90·5–92·0)	0·141 (0·042–0·275)	0·137 (0·040–0·270)	-2·9 (-7·1–-0·8)
Singapore	90·7 (90·0–91·5)	0·141 (0·042–0·275)	0·138 (0·040–0·272)	-2·4 (-5·7–-0·6)
**High-income North America**	**87·6 (86·8–88·6)**	**0·141 (0·042–0·275)**	**0·141 (0·042–0·275)**	**0·5 (-0·5–1·8)**

Canada	94·5 (94·0–95·0)	0·141 (0·042–0·275)	0·134 (0·038–0·263)	-5·7 (-13·6—1·7)
Greenland	63·6 (62·0–65·0)	0·141 (0·042–0·275)	0·167 (0·058–0·314)	21·5 (6·4–49·6)
USA	86·9 (86·0–88·0)	0·141 (0·042–0·275)	0·142 (0·042–0·276)	1·0 (-0·0–2·8)
**Southern Latin America**	**70·1 (69·1–71·1)**	**0·141 (0·042–0·275)**	**0·160 (0·054–0·302)**	**15·5 (4·7–36·0)**
Argentina	67·0 (65·5–68·5)	0·141 (0·042–0·275)	0·164 (0·056–0·308)	18·5 (5·5–42·6)
Chile	77·7 (76·5–79·0)	0·141 (0·042–0·275)	0·152 (0·049–0·288)	9·1 (2·7–20·6)
Uruguay	70·7 (69·5–72·0)	0·141 (0·042–0·275)	0·160 (0·053–0·302)	15·2 (4·6–35·2)
**Western Europe**	**93·9 (93·7–94·2)**	**0·141 (0·042–0·275)**	**0·134 (0·038–0·264)**	**-5·2 (-12·3–-1·5)**
Andorra	95·8 (95·0–96·5)	0·141 (0·042–0·275)	0·133 (0·037–0·260)	-6·8 (-15·8—2·0)
Austria	93·7 (93·0–94·5)	0·141 (0·042–0·275)	0·135 (0·038–0·265)	-5·0 (-11·6—1·4)
Belgium	93·5 (93·0–94·5)	0·141 (0·042–0·275)	0·135 (0·038–0·265)	-4·8 (-11·3—1·4)
Cyprus	93·1 (92·5–94·0)	0·141 (0·042–0·275)	0·135 (0·038–0·267)	-4·5 (-10·6—1·3)
Denmark	92·6 (92·0–93·5)	0·141 (0·042–0·275)	0·136 (0·039–0·267)	-4·1 (-9·7—1·1)
Finland	94·3 (93·5–95·0)	0·141 (0·042–0·275)	0·134 (0·038–0·263)	-5·6 (-13·3—1·6)
France	94·2 (93·5–95·0)	0·141 (0·042–0·275)	0·134 (0·037–0·264)	-5·4 (-12·4—1·6)
Germany	92·6 (92·0–93·5)	0·141 (0·042–0·275)	0·136 (0·039–0·267)	-4·0 (-9·7—1·1)
Greece	91·6 (91·0–92·5)	0·141 (0·042–0·275)	0·137 (0·039–0·270)	-3·2 (-7·7—0·9)
Iceland	96·7 (96·0–97·5)	0·141 (0·042–0·275)	0·132 (0·036–0·258)	-7·7 (-18·4—2·3)
Ireland	96·6 (96·0–97·0)	0·141 (0·042–0·275)	0·132 (0·036–0·258)	-7·6 (-17·7—2·2)
Israel	88·5 (87·5–89·5)	0·141 (0·042–0·275)	0·140 (0·041–0·274)	-0·4 (-1·9–0·5)
Italy	96·1 (95·5–96·5)	0·141 (0·042–0·275)	0·132 (0·036–0·260)	-7·1 (-16·5—2·1)
Luxembourg	95·2 (94·5–96·0)	0·141 (0·042–0·275)	0·133 (0·037–0·261)	-6·4 (-15·0—1·8)
Malta	91·1 (90·5–92·0)	0·141 (0·042–0·275)	0·138 (0·040–0·270)	-2·7 (-6·6—0·8)
Monaco	95·8 (94·5–97·0)	0·141 (0·042–0·275)	0·133 (0·036–0·260)	-6·8 (-15·4—2·0)
Netherlands	96·8 (96·5–97·5)	0·141 (0·042–0·275)	0·131 (0·036–0·258)	-7·7 (-18·2—2·3)
Norway	96·7 (96·0–97·5)	0·141 (0·042–0·275)	0·132 (0·036–0·259)	-7·6 (-17·7—2·2)
Portugal	90·5 (89·5–91·5)	0·141 (0·042–0·275)	0·138 (0·040–0·271)	-2·2 (-5·5—0·6)
San Marino	95·2 (93·5–96·5)	0·141 (0·042–0·275)	0·133 (0·037–0·262)	-6·3 (-15·1—1·8)
Spain	95·7 (95·0–96·5)	0·141 (0·042–0·275)	0·133 (0·037–0·260)	-6·7 (-15·6—2·0)
Sweden	95·9 (95·0–96·5)	0·141 (0·042–0·275)	0·132 (0·037–0·260)	-6·9 (-15·6—2·0)
Switzerland	96·2 (95·5–96·5)	0·141 (0·042–0·275)	0·132 (0·037–0·260)	-7·2 (-17·0—2·1)
UK	92·3 (91·5–93·0)	0·141 (0·042–0·275)	0·136 (0·039–0·268)	-3·8 (-8·9—1·1)
**Latin America and Caribbean**	**61·3 (60·7–62·0)**	**0·141 (0·042–0·275)**	**0·169 (0·060–0·316)**	**23·2 (6·9–53·1)**
**Andean Latin America**	**59·2 (58·3–60·2)**	**0·141 (0·042–0·275)**	**0·172 (0·061–0·320)**	**25·1 (7·4–58·2)**
Bolivia	46·6 (45·0–48·0)	0·141 (0·042–0·275)	0·185 (0·069–0·339)	35·7 (10·6–81·9)
Ecuador	59·5 (58·0–61·0)	0·141 (0·042–0·275)	0·172 (0·061–0·321)	25·1 (7·4–58·7)
Peru	63·5 (62·0–65·0)	0·141 (0·042–0·275)	0·167 (0·058–0·313)	21·6 (6·3–49·6)
**Caribbean**	**55·0 (54·4–55·7)**	**0·141 (0·042–0·275)**	**0·171 (0·060–0·320)**	**24·8 (7·3–56·0)**
Antigua and Barbuda	65·2 (64·0–66·5)	0·141 (0·042–0·275)	0·166 (0·056–0·310)	20·0 (5·9–46·6)
The Bahamas	59·5 (58·0–61·0)	0·141 (0·042–0·275)	0·172 (0·060–0·320)	25·0 (7·4–57·8)
Barbados	67·0 (65·5–68·5)	0·141 (0·042–0·275)	0·164 (0·056–0·307)	18·4 (5·4–42·8)
Belize	54·4 (53·0–56·0)	0·141 (0·042–0·275)	0·177 (0·064–0·328)	29·6 (8·7–68·1)
Bermuda	83·6 (82·5–84·5)	0·141 (0·042–0·275)	0·146 (0·044–0·280)	3·9 (1·1–9·1)
Cuba	74·4 (73·0–76·0)	0·141 (0·042–0·275)	0·156 (0·051–0·294)	11·9 (3·5–28·0)
Dominica	53·1 (51·5–54·5)	0·141 (0·042–0·275)	0·179 (0·065–0·329)	30·7 (9·1–71·0)
Dominican Republic	54·6 (53·0–56·0)	0·141 (0·042–0·275)	0·177 (0·064–0·328)	29·3 (8·7–67·1)

Grenada	57·8 (56·5–59·5)	0·141 (0·042–0·275)	0·174 (0·061–0·321)	26·5 (8·0–61·8)
Guyana	46·5 (45·0–48·0)	0·141 (0·042–0·275)	0·185 (0·069–0·340)	35·7 (10·6–81·8)
Haiti	28·7 (27·0–30·0)	0·141 (0·042–0·275)	0·188 (0·070–0·341)	38·2 (11·4–84·3)
Jamaica	61·2 (59·5–62·5)	0·141 (0·042–0·275)	0·170 (0·059–0·316)	23·5 (6·9–53·7)
Puerto Rico	79·5 (78·5–81·0)	0·141 (0·042–0·275)	0·150 (0·047–0·284)	7·4 (2·1–16·9)
Saint Kitts and Nevis	67·5 (60·0–75·5)	0·141 (0·042–0·275)	0·163 (0·056–0·307)	18·0 (4·6–41·4)
Saint Lucia	60·5 (59·0–62·0)	0·141 (0·042–0·275)	0·171 (0·059–0·318)	24·2 (7·4–56·3)
Saint Vincent and the Grenadines	54·1 (52·5–55·5)	0·141 (0·042–0·275)	0·178 (0·064–0·328)	29·8 (8·8–68·3)
Suriname	50·4 (49·0–52·0)	0·141 (0·042–0·275)	0·182 (0·067–0·334)	33·0 (9·7–76·1)
Trinidad and Tobago	58·8 (57·5–60·0)	0·141 (0·042–0·275)	0·173 (0·061–0·321)	25·7 (7·6–59·8)
Virgin Islands	65·2 (63·5–66·5)	0·141 (0·042–0·275)	0·166 (0·056–0·310)	20·1 (6·0–46·7)
**Central Latin America**	**63·2 (62·4–64·0)**	**0·141 (0·042–0·275)**	**0·167 (0·058–0·313)**	**21·6 (6·4–50·7)**
Colombia	69·3 (68·0–70·5)	0·141 (0·042–0·275)	0·161 (0·054–0·303)	16·4 (4·8–37·7)
Costa Rica	73·6 (72·0–75·0)	0·141 (0·042–0·275)	0·157 (0·052–0·297)	12·7 (3·7–29·2)
El Salvador	62·0 (60·5–63·5)	0·141 (0·042–0·275)	0·169 (0·059–0·316)	22·8 (6·8–52·5)
Guatemala	49·5 (48·0–51·0)	0·141 (0·042–0·275)	0·182 (0·068–0·335)	33·7 (10·1–78·1)
Honduras	49·3 (47·5–51·0)	0·141 (0·042–0·275)	0·183 (0·068–0·336)	33·9 (10·1–79·5)
Mexico	63·0 (61·5–64·5)	0·141 (0·042–0·275)	0·168 (0·059–0·314)	22·0 (6·6–50·4)
Nicaragua	63·5 (62·0–65·0)	0·141 (0·042–0·275)	0·167 (0·058–0·313)	21·5 (6·3–50·1)
Panama	68·3 (67·0–69·5)	0·141 (0·042–0·275)	0·162 (0·055–0·304)	17·3 (5·2–40·3)
Venezuela	64·8 (63·0–66·5)	0·141 (0·042–0·275)	0·166 (0·058–0·312)	20·4 (6·0–47·8)
**Tropical Latin America**	**61·2 (59·9–62·4)**	**0·141 (0·042–0·275)**	**0·170 (0·060–0·317)**	**23·5 (7·0–53·7)**
Brazil	61·3 (60·0–62·5)	0·141 (0·042–0·275)	0·170 (0·060–0·317)	23·5 (7·0–53·7)
Paraguay	59·4 (58·0–61·0)	0·141 (0·042–0·275)	0·172 (0·060–0·320)	25·1 (7·4–56·9)
**North Africa and Middle East**	**61·3 (60·8–61·8)**	**0·141 (0·042–0·275)**	**0·167 (0·058–0·314)**	**21·6 (6·3–50·1)**
Afghanistan	26·9 (25·5–28·5)	0·141 (0·042–0·275)	0·188 (0·070–0·341)	38·2 (11·4–84·3)
Algeria	65·9 (64·5–67·5)	0·141 (0·042–0·275)	0·165 (0·056–0·309)	19·4 (5·8–45·6)
Bahrain	74·5 (73·0–76·0)	0·141 (0·042–0·275)	0·156 (0·051–0·292)	11·9 (3·6–27·6)
Egypt	56·9 (55·5–58·5)	0·141 (0·042–0·275)	0·175 (0·063–0·323)	27·3 (8·0–64·8)
Iran	70·9 (69·5–72·5)	0·141 (0·042–0·275)	0·159 (0·053–0·300)	15·0 (4·3–34·8)
Iraq	61·9 (60·0–63·5)	0·141 (0·042–0·275)	0·169 (0·059–0·316)	23·0 (6·8–53·6)
Jordan	70·8 (69·5–72·5)	0·141 (0·042–0·275)	0·160 (0·054–0·300)	15·1 (4·4–34·4)
Kuwait	82·5 (81·0–84·0)	0·141 (0·042–0·275)	0·147 (0·045–0·280)	4·8 (1·4–11·2)
Lebanon	81·1 (80·0–82·5)	0·141 (0·042–0·275)	0·148 (0·046–0·281)	6·0 (1·8–13·5)
Libya	63·1 (61·5–64·5)	0·141 (0·042–0·275)	0·168 (0·058–0·313)	21·9 (6·5–50·6)
Morocco	54·8 (53·0–56·5)	0·141 (0·042–0·275)	0·177 (0·064–0·328)	29·2 (8·6–68·1)
Oman	76·1 (74·5–77·5)	0·141 (0·042–0·275)	0·154 (0·050–0·291)	10·5 (3·0–24·3)
Palestine	63·3 (61·5–65·0)	0·141 (0·042–0·275)	0·168 (0·057–0·314)	21·7 (6·3–49·6)
Qatar	81·2 (80·0–82·5)	0·141 (0·042–0·275)	0·148 (0·046–0·282)	5·9 (1·8–13·8)
Saudi Arabia	74·3 (73·0–76·0)	0·141 (0·042–0·275)	0·156 (0·051–0·294)	12·1 (3·6–28·2)
Sudan	47·2 (45·5–49·0)	0·141 (0·042–0·275)	0·184 (0·068–0·338)	35·3 (10·5–81·8)
Syria	65·3 (64·0–67·0)	0·141 (0·042–0·275)	0·165 (0·056–0·311)	19·9 (6·0–44·9)
Tunisia	73·2 (72·0–74·5)	0·141 (0·042–0·275)	0·157 (0·052–0·297)	13·0 (3·8–29·2)
Turkey	73·5 (72·0–75·0)	0·141 (0·042–0·275)	0·157 (0·052–0·296)	12·8 (3·8–29·5)
United Arab Emirates	61·2 (59·5–63·0)	0·141 (0·042–0·275)	0·170 (0·060–0·316)	23·6 (7·1–54·6)
Yemen	42·9 (41·0–44·5)	0·141 (0·042–0·275)	0·187 (0·070–0·341)	37·3 (10·9–84·0)

**South Asia**	**40·3 (39·1–41·6)**	**0·141 (0·042–0·275)**	**0·187 (0·070–0·341)**	**37·7 (11·2–83·7)**
Bangladesh	46·1 (44·5–47·5)	0·141 (0·042–0·275)	0·185 (0·069–0·339)	36·0 (10·7–81·9)
Bhutan	49·6 (48·0–51·0)	0·141 (0·042–0·275)	0·182 (0·068–0·335)	33·6 (9·9–78·4)
India	40·4 (39·0–42·0)	0·141 (0·042–0·275)	0·187 (0·070–0·341)	37·8 (11·2–84·0)
Nepal	41·5 (40·0–43·0)	0·141 (0·042–0·275)	0·187 (0·070–0·341)	37·6 (11·2–84·0)
Pakistan	35·6 (34·0–37·0)	0·141 (0·042–0·275)	0·188 (0·070–0·341)	38·1 (11·3–84·3)
**Southeast Asia, east Asia, and Oceania**	**70·6 (69·7–71·5)**	**0·141 (0·042–0·275)**	**0·160 (0·053–0·302)**	**15·6 (4·7–35·6)**
**East Asia**	**79·5 (78·2–80·6)**	**0·141 (0·042–0·275)**	**0·150 (0·047–0·284)**	**7·5 (2·3–17·5)**
China	79·9 (78·5–81·0)	0·141 (0·042–0·275)	0·150 (0·047–0·283)	7·2 (2·1–16·8)
North Korea	54·7 (53·0–56·0)	0·141 (0·042–0·275)	0·177 (0·064–0·329)	29·3 (8·7–68·2)
Taiwan (province of China)	86·7 (85·5–88·0)	0·141 (0·042–0·275)	0·142 (0·042–0·276)	1·1 (0·1–3·4)
**Oceania**	**34·2 (33·0–35·3)**	**0·141 (0·042–0·275)**	**0·187 (0·069–0·341)**	**37·5 (11·1–83·1)**
American Samoa	52·5 (51·0–54·0)	0·141 (0·042–0·275)	0·179 (0·065–0·331)	31·2 (9·1–71·4)
Cook Islands	70·1 (63·0–78·0)	0·141 (0·042–0·275)	0·160 (0·054–0·301)	15·9 (3·8–39·7)
Fiji	44·7 (43·0–46·0)	0·141 (0·042–0·275)	0·186 (0·070–0·341)	36·6 (10·8–82·2)
Guam	62·2 (61·0–63·5)	0·141 (0·042–0·275)	0·169 (0·058–0·316)	22·7 (6·8–53·8)
Kiribati	28·2 (27·0–29·5)	0·141 (0·042–0·275)	0·188 (0·070–0·341)	38·2 (11·4–84·3)
Marshall Islands	36·4 (35·0–38·0)	0·141 (0·042–0·275)	0·188 (0·070–0·341)	38·1 (11·3–84·3)
Federated States of Micronesia	43·1 (41·5–44·5)	0·141 (0·042–0·275)	0·187 (0·070–0·341)	37·2 (11·2–84·0)
Nauru	51·8 (43·0–60·0)	0·141 (0·042–0·275)	0·180 (0·067–0·334)	31·6 (9·0–73·0)
Niue	64·4 (56·0–73·0)	0·141 (0·042–0·275)	0·166 (0·059–0·306)	20·8 (5·8–50·1)
Northern Mariana Islands	68·7 (67·5–70·0)	0·141 (0·042–0·275)	0·162 (0·054–0·305)	17·0 (5·0–38·8)
Palau	64·2 (56·0–72·5)	0·141 (0·042–0·275)	0·167 (0·057–0·312)	20·9 (5·8–50·1)
Papua New Guinea	31·5 (30·0–33·0)	0·141 (0·042–0·275)	0·188 (0·070–0·341)	38·2 (11·4–84·3)
Samoa	50·5 (49·0–52·0)	0·141 (0·042–0·275)	0·181 (0·067–0·334)	32·9 (9·7–76·2)
Solomon Islands	39·6 (38·0–41·0)	0·141 (0·042–0·275)	0·188 (0·070–0·341)	37·9 (11·2–84·0)
Tokelau	54·4 (46·0–63·0)	0·141 (0·042–0·275)	0·177 (0·065–0·326)	29·5 (8·4–65·8)
Tonga	49·0 (47·5–50·5)	0·141 (0·042–0·275)	0·183 (0·068–0·336)	34·1 (10·3–78·4)
Tuvalu	45·9 (38·0–54·0)	0·141 (0·042–0·275)	0·185 (0·068–0·337)	35·5 (10·5–78·7)
Vanuatu	32·8 (31·5–34·0)	0·141 (0·042–0·275)	0·188 (0·070–0·341)	38·2 (11·4–84·3)
**Southeast Asia**	**51·8 (51·0–52·5)**	**0·141 (0·042–0·275)**	**0·179 (0·065–0·332)**	**31·2 (9·3–70·8)**
Cambodia	44·4 (43·0–46·0)	0·141 (0·042–0·275)	0·186 (0·070–0·341)	36·8 (10·9–84·0)
Indonesia	44·7 (43·0–46·0)	0·141 (0·042–0·275)	0·186 (0·069–0·340)	36·6 (10·9–83·0)
Laos	36·6 (35·0–38·0)	0·141 (0·042–0·275)	0·188 (0·070–0·341)	38·1 (11·3–84·3)
Malaysia	65·5 (64·0–67·0)	0·141 (0·042–0·275)	0·165 (0·057–0·309)	19·8 (5·8–45·3)
Maldives	71·8 (70·5–73·0)	0·141 (0·042–0·275)	0·158 (0·053–0·299)	14·2 (4·2–32·9)
Mauritius	65·9 (64·5–67·5)	0·141 (0·042–0·275)	0·165 (0·056–0·311)	19·4 (5·8–44·1)
Myanmar	42·6 (41·0–44·0)	0·141 (0·042–0·275)	0·187 (0·070–0·341)	37·4 (11·1–84·0)
Philippines	46·5 (45·0–48·0)	0·141 (0·042–0·275)	0·185 (0·068–0·339)	35·7 (10·8–81·8)
Seychelles	61·6 (60·0–63·0)	0·141 (0·042–0·275)	0·169 (0·059–0·315)	23·2 (7·0–52·7)
Sri Lanka	69·7 (68·5–71·0)	0·141 (0·042–0·275)	0·161 (0·054–0·303)	16·1 (4·7–36·5)
Thailand	69·3 (68·0–70·5)	0·141 (0·042–0·275)	0·161 (0·054–0·303)	16·4 (5·0–38·3)
Timor-Leste	40·0 (38·5–41·5)	0·141 (0·042–0·275)	0·187 (0·070–0·341)	37·8 (11·2–84·0)
Vietnam	63·1 (61·5–64·5)	0·141 (0·042–0·275)	0·168 (0·058–0·315)	21·9 (6·6–50·3)
**Sub-Saharan Africa**	**32·3 (31·9–32·8)**	**0·141 (0·042–0·275)**	**0·188 (0·070–0·341)**	**37·9 (11·3–84·3)**
**Central sub-Saharan Africa**	**27·2 (26·2–28·2)**	**0·141 (0·042–0·275)**	**0·188 (0·070–0·341)**	**38·2 (11·4–84·3)**

Angola	30·3 (29·0–31·5)	0·141 (0·042–0·275)	0·188 (0·070–0·341)	38·2 (11·4–84·3)
Central African Republic	12·8 (12·0–14·0)	0·141 (0·042–0·275)	0·188 (0·070–0·341)	38·2 (11·4–84·3)
Congo (Brazzaville)	32·5 (31·0–34·0)	0·141 (0·042–0·275)	0·188 (0·070–0·341)	38·2 (11·4–84·3)
DR Congo	26·2 (25·0–27·5)	0·141 (0·042–0·275)	0·188 (0·070–0·341)	38·2 (11·4–84·3)
Equatorial Guinea	42·4 (41·0–44·0)	0·141 (0·042–0·275)	0·187 (0·070–0·341)	37·4 (11·2–84·0)
Gabon	41·0 (39·5–42·5)	0·141 (0·042–0·275)	0·187 (0·070–0·341)	37·7 (11·2–84·0)
**Eastern sub-Saharan Africa**	**30·8 (30·3–31·4)**	**0·141 (0·042–0·275)**	**0·188 (0·070–0·341)**	**38·2 (11·3–84·3)**
Burundi	25·6 (24·5–27·0)	0·141 (0·042–0·275)	0·188 (0·070–0·341)	38·2 (11·4–84·3)
Comoros	31·9 (30·5–33·5)	0·141 (0·042–0·275)	0·188 (0·070–0·341)	38·2 (11·4–84·3)
Djibouti	36·4 (35·0–38·0)	0·141 (0·042–0·275)	0·188 (0·070–0·341)	38·1 (11·3–84·3)
Eritrea	25·4 (24·0–26·5)	0·141 (0·042–0·275)	0·188 (0·070–0·341)	38·2 (11·4–84·3)
Ethiopia	31·9 (30·5–33·5)	0·141 (0·042–0·275)	0·188 (0·070–0·341)	38·2 (11·3–84·3)
Kenya	34·3 (33·0–36·0)	0·141 (0·042–0·275)	0·188 (0·070–0·341)	38·1 (11·3–84·3)
Madagascar	26·8 (25·5–28·0)	0·141 (0·042–0·275)	0·188 (0·070–0·341)	38·2 (11·4–84·3)
Malawi	30·0 (28·5–31·5)	0·141 (0·042–0·275)	0·188 (0·070–0·341)	38·2 (11·4–84·3)
Mozambique	27·2 (26·0–28·5)	0·141 (0·042–0·275)	0·188 (0·070–0·341)	38·2 (11·4–84·3)
Rwanda	34·5 (33·0–36·0)	0·141 (0·042–0·275)	0·188 (0·070–0·341)	38·1 (11·4–84·3)
Somalia	16·1 (15·0–17·0)	0·141 (0·042–0·275)	0·188 (0·070–0·341)	38·2 (11·4–84·3)
South Sudan	21·7 (20·5–23·0)	0·141 (0·042–0·275)	0·188 (0·070–0·341)	38·2 (11·4–84·3)
Uganda	33·5 (32·0–35·0)	0·141 (0·042–0·275)	0·188 (0·070–0·341)	38·1 (11·3–84·3)
Tanzania	34·1 (32·5–35·5)	0·141 (0·042–0·275)	0·188 (0·070–0·341)	38·1 (11·3–84·3)
Zambia	35·5 (34·0–37·0)	0·141 (0·042–0·275)	0·188 (0·070–0·341)	38·1 (11·3–84·3)
**Southern sub-Saharan Africa**	**43·3 (42·1–44·4)**	**0·141 (0·042–0·275)**	**0·185 (0·069–0·339)**	**35·9 (10·8–82·2)**
Botswana	50·8 (49·0–52·5)	0·141 (0·042–0·275)	0·181 (0·066–0·333)	32·6 (9·8–76·1)
eSwatini	36·2 (35·0–37·5)	0·141 (0·042–0·275)	0·188 (0·070–0·341)	38·1 (11·3–84·3)
Lesotho	29·7 (28·5–31·0)	0·141 (0·042–0·275)	0·188 (0·070–0·341)	38·2 (11·4–84·3)
Namibia	44·8 (43·5–46·5)	0·141 (0·042–0·275)	0·186 (0·070–0·340)	36·6 (10·9–83·0)
South Africa	46·9 (45·5–48·5)	0·141 (0·042–0·275)	0·185 (0·069–0·338)	35·5 (10·7–81·8)
Zimbabwe	30·8 (29·0–32·0)	0·141 (0·042–0·275)	0·188 (0·070–0·341)	38·2 (11·4–84·3)
**Western sub-Saharan Africa**	**33·3 (32·6–34·1)**	**0·141 (0·042–0·275)**	**0·188 (0·070–0·341)**	**38·1 (11·3–84·3)**
Benin	31·3 (30·0–32·5)	0·141 (0·042–0·275)	0·188 (0·070–0·341)	38·2 (11·4–84·3)
Burkina Faso	30·5 (29·0–32·0)	0·141 (0·042–0·275)	0·188 (0·070–0·341)	38·2 (11·4–84·3)
Cape Verde	56·3 (54·5–58·0)	0·141 (0·042–0·275)	0·175 (0·064–0·325)	27·9 (8·3–64·6)
Cameroon	35·4 (34·0–37·0)	0·141 (0·042–0·275)	0·188 (0·070–0·341)	38·1 (11·3–84·3)
Chad	23·2 (22·0–24·5)	0·141 (0·042–0·275)	0·188 (0·070–0·341)	38·2 (11·4–84·3)
Côte d'Ivoire	30·5 (29·0–32·0)	0·141 (0·042–0·275)	0·188 (0·070–0·341)	38·2 (11·4–84·3)
The Gambia	33·2 (31·5–35·0)	0·141 (0·042–0·275)	0·188 (0·070–0·341)	38·2 (11·4–84·3)
Ghana	39·4 (38·0–41·0)	0·141 (0·042–0·275)	0·188 (0·070–0·341)	37·9 (11·2–84·0)
Guinea	25·4 (24·0–26·5)	0·141 (0·042–0·275)	0·188 (0·070–0·341)	38·2 (11·4–84·3)
Guinea-Bissau	24·3 (23·0–25·5)	0·141 (0·042–0·275)	0·188 (0·070–0·341)	38·2 (11·4–84·3)
Liberia	32·3 (30·5–34·0)	0·141 (0·042–0·275)	0·188 (0·070–0·341)	38·2 (11·4–84·3)
Mali	30·0 (28·5–31·5)	0·141 (0·042–0·275)	0·188 (0·070–0·341)	38·2 (11·4–84·3)
Mauritania	40·4 (39·0–42·0)	0·141 (0·042–0·275)	0·187 (0·070–0·341)	37·8 (11·2–84·0)
Niger	26·7 (25·5–28·0)	0·141 (0·042–0·275)	0·188 (0·070–0·341)	38·2 (11·4–84·3)
Nigeria	35·2 (33·5–36·5)	0·141 (0·042–0·275)	0·188 (0·070–0·341)	38·1 (11·3–84·3)
São Tomé and PrÍncipe	42·6 (41·0–44·0)	0·141 (0·042–0·275)	0·187 (0·070–0·341)	37·4 (10·9–84·0)

Senegal	32·5 (31·0–34·0)	0·141 (0·042–0·275)	0·188 (0·070–0·341)	38·2 (11·4–84·3)
Sierra Leone	30·5 (29·0–32·0)	0·141 (0·042–0·275)	0·188 (0·070–0·341)	38·2 (11·4–84·3)
Togo	33·9 (32·5–35·5)	0·141 (0·042–0·275)	0·188 (0·070–0·341)	38·1 (11·3–84·3)

HAQI = Healthcare access quality index. DW = Disability weight. Numbers in parentheses represent 95% uncertainty intervals. HAQI estimates for regions, super-regions, and the globe were calculated by population-weighting country-specific HAQI estimates.

Table S4: Severity proportions for anxiety disorders for 2019 by country, region, and super-region

Location	Asymptomatic	Mild	Moderate	Severe
**Global**	**9·6 (1·7–22·8)**	**37·8 (15·6–59·8)**	**29·7 (12·6–42·6)**	**22·9 (4·5–53·0)**
**Central Europe, eastern Europe, and central Asia**	**15·2 (2·6–36·2)**	**34·3 (15·6–49·5)**	**29·5 (12·9–42·6)**	**21·0 (3·9–48·4)**
**Central Asia**	**8·3 (1·3–19·8)**	**38·6 (15·4–63·2)**	**29·7 (12·6–42·5)**	**23·4 (4·7–53·8)**
Armenia	13·4 (2·4–32·1)	35·4 (15·5–52·7)	29·6 (12·8–42·5)	21·6 (4·1–50·7)
Azerbaijan	8·1 (1·3–19·8)	38·7 (15·4–63·3)	29·7 (12·6–42·6)	23·5 (4·7–54·3)
Georgia	10·2 (1·8–25·2)	37·4 (15·6–59·4)	29·7 (12·7–42·5)	22·7 (4·4–52·6)
Kazakhstan	12·2 (2·0–29·0)	36·2 (15·7–55·2)	29·6 (12·7–42·5)	22·0 (4·1–50·7)
Kyrgyzstan	8·1 (1·3–19·8)	38·7 (15·4–63·6)	29·7 (12·6–42·4)	23·4 (4·8–53·9)
Mongolia	3·8 (0·0–12·0)	41·4 (15·0–72·7)	29·9 (12·7–43·1)	24·9 (5·3–56·4)
Tajikistan	2·2 (0·0–8·7)	42·4 (14·9–76·3)	29·9 (12·6–43·2)	25·5 (5·4–57·1)
Turkmenistan	7·5 (1·2–18·3)	39·1 (15·4–64·9)	29·8 (12·6–42·7)	23·6 (4·9–54·5)
Uzbekistan	7·9 (1·3–18·9)	38·8 (15·4–64·0)	29·7 (12·6–42·5)	23·5 (4·7–53·8)
**Central Europe**	**19·0 (3·3–45·5)**	**32·0 (15·9–43·9)**	**29·4 (12·8–42·9)**	**19·6 (3·6–46·8)**
Albania	17·7 (3·1–42·0)	32·8 (15·7–45·7)	29·4 (12·8–42·6)	20·1 (3·7–47·0)
Bosnia and Herzegovina	15·8 (2·7–37·6)	34·0 (15·6–48·7)	29·5 (12·9–42·5)	20·7 (3·8–48·1)
Bulgaria	15·5 (2·7–36·8)	34·1 (15·6–49·2)	29·5 (12·9–42·5)	20·9 (3·8–48·1)
Croatia	23·2 (4·1–55·6)	29·4 (16·1–39·4)	29·2 (12·3–43·7)	18·2 (3·0–45·1)
Czech Republic	22·7 (4·0–54·4)	29·7 (16·1–39·5)	29·3 (12·2–43·7)	18·3 (3·1–45·3)
Hungary	20·0 (3·5–47·6)	31·3 (15·9–42·7)	29·4 (12·6–42·9)	19·3 (3·4–46·5)
Montenegro	19·3 (3·4–46·2)	31·8 (15·8–43·8)	29·4 (12·7–42·9)	19·5 (3·5–46·6)
North Macedonia	16·3 (2·8–39·1)	33·6 (15·6–47·5)	29·5 (12·9–42·5)	20·6 (3·7–47·9)
Poland	19·2 (3·4–46·2)	31·9 (16·0–43·8)	29·4 (12·7–42·9)	19·6 (3·5–46·7)
Romania	17·1 (3·0–40·8)	33·2 (15·7–46·5)	29·4 (13·0–42·6)	20·3 (3·7–47·2)
Serbia	18·0 (3·2–42·9)	32·6 (15·8–45·4)	29·4 (13·0–42·7)	20·0 (3·6–47·0)
Slovakia	20·2 (3·5–47·9)	31·2 (15·9–42·6)	29·3 (12·5–43·2)	19·2 (3·4–46·2)
Slovenia	24·9 (4·4–59·6)	28·3 (15·8–37·9)	29·2 (12·2–44·2)	17·6 (2·7–43·6)
**Eastern Europe**	**16·1 (2·8–38·1)**	**33·7 (15·6–48·1)**	**29·5 (12·9–42·6)**	**20·6 (3·8–47·6)**
Belarus	18·8 (3·3–44·3)	32·1 (15·9–44·3)	29·4 (12·7–42·9)	19·7 (3·6–46·8)
Estonia	20·9 (3·7–49·8)	30·8 (16·0–41·8)	29·3 (12·5–43·3)	19·0 (3·3–46·2)
Latvia	17·3 (3·0–40·9)	33·0 (15·7–46·6)	29·4 (13·0–42·6)	20·2 (3·6–47·5)
Lithuania	16·5 (2·9–39·6)	33·5 (15·7–47·4)	29·5 (12·9–42·6)	20·5 (3·7–47·8)
Moldova	12·3 (2·0–29·8)	36·1 (15·6–55·0)	29·6 (12·7–42·5)	21·9 (4·1–50·8)
Russia	16·4 (2·9–39·0)	33·6 (15·6–47·5)	29·5 (12·9–42·6)	20·5 (3·8–47·3)
Ukraine	15·0 (2·6–35·3)	34·5 (15·6–50·4)	29·5 (12·9–42·5)	21·0 (3·9–48·9)
**High income**	**24·1 (4·3–58·0)**	**28·8 (16·0–38·6)**	**29·2 (12·2–44·0)**	**17·8 (2·9–44·3)**
**Australasia**	**26·5 (4·7–63·6)**	**27·4 (15·0–37·0)**	**29·1 (12·2–44·7)**	**17·0 (2·5–42·7)**
Australia	26·7 (4·8–64·1)	27·2 (14·8–36·9)	29·1 (12·2–44·8)	16·9 (2·4–42·4)
New Zealand	25·0 (4·4–59·6)	28·3 (15·7–37·8)	29·2 (12·2–44·3)	17·6 (2·7–43·8)
**High-income Asia Pacific**	**25·6 (4·6–61·6)**	**27·9 (15·7–37·4)**	**29·2 (12·2–44·5)**	**17·3 (2·6–43·4)**
Brunei	12·6 (2·2–30·4)	36·0 (15·6–54·5)	29·6 (12·7–42·5)	21·9 (4·1–50·2)
Japan	26·1 (4·6–62·7)	27·6 (15·4–37·0)	29·2 (12·2–44·5)	17·2 (2·5–43·0)
South Korea	24·7 (4·4–59·1)	28·4 (15·8–37·9)	29·2 (12·2–44·2)	17·6 (2·7–44·0)
Singapore	24·4 (4·4–58·9)	28·6 (15·9–38·2)	29·2 (12·2–44·0)	17·7 (2·8–44·1)
**High-income North America**	**22·8 (4·1–54·6)**	**29·6 (16·1–39·8)**	**29·3 (12·3–43·7)**	**18·3 (3·1–45·2)**

Canada	26·4 (4·7–63·3)	27·4 (14·9–37·0)	29·1 (12·2–44·7)	17·1 (2·5–42·8)
Greenland	10·1 (1·7–24·6)	37·5 (15·5–59·3)	29·7 (12·7–42·6)	22·8 (4·5–52·5)
USA	22·4 (4·0–53·5)	29·9 (16·1–40·2)	29·3 (12·3–43·7)	18·5 (3·2–45·4)
**Southern Latin America**	**13·5 (2·3–32·1)**	**35·4 (15·6–52·6)**	**29·6 (12·8–42·5)**	**21·5 (4·0–50·0)**
Argentina	11·9 (2·0–28·0)	36·4 (15·7–56·6)	29·6 (12·7–42·5)	22·1 (4·2–51·3)
Chile	17·5 (3·0–41·6)	32·9 (15·7–46·0)	29·4 (13·0–42·6)	20·2 (3·7–47·1)
Uruguay	13·9 (2·4–33·1)	35·2 (15·6–52·0)	29·5 (12·8–42·5)	21·4 (4·0–49·7)
**Western Europe**	**26·1 (4·6–62·9)**	**27·6 (15·6–37·2)**	**29·2 (12·2–44·6)**	**17·2 (2·5–42·9)**
Andorra	27·1 (4·9–65·0)	27·0 (14·5–36·8)	29·1 (12·2–44·9)	16·8 (2·4–42·1)
Austria	26·0 (4·6–62·4)	27·7 (15·5–37·2)	29·2 (12·2–44·5)	17·2 (2·6–43·0)
Belgium	25·9 (4·6–62·1)	27·7 (15·6–37·1)	29·2 (12·2–44·5)	17·2 (2·6–43·0)
Cyprus	25·7 (4·6–61·2)	27·8 (15·7–37·5)	29·2 (12·2–44·4)	17·3 (2·6–43·4)
Denmark	25·4 (4·5–61·5)	28·0 (15·7–37·5)	29·2 (12·2–44·3)	17·4 (2·6–43·4)
Finland	26·3 (4·7–62·8)	27·4 (15·0–37·1)	29·1 (12·2–44·6)	17·1 (2·5–42·8)
France	26·2 (4·6–62·8)	27·5 (15·1–37·1)	29·2 (12·2–44·7)	17·1 (2·5–42·8)
Germany	25·4 (4·5–61·0)	28·0 (15·7–37·6)	29·2 (12·2–44·3)	17·4 (2·6–43·4)
Greece	24·9 (4·4–59·8)	28·3 (15·8–37·9)	29·2 (12·2–44·2)	17·6 (2·7–44·0)
Iceland	27·6 (4·9–65·9)	26·7 (14·1–36·6)	29·1 (12·1–45·3)	16·6 (2·3–41·7)
Ireland	27·5 (5·0–65·9)	26·7 (14·2–36·7)	29·1 (12·2–45·2)	16·7 (2·3–41·9)
Israel	23·2 (4·1–55·1)	29·4 (16·1–39·4)	29·2 (12·2–43·9)	18·2 (3·0–44·7)
Italy	27·3 (4·9–65·2)	26·9 (14·5–36·7)	29·1 (12·2–45·1)	16·8 (2·4–42·1)
Luxembourg	26·8 (4·8–64·4)	27·2 (14·9–36·9)	29·1 (12·2–44·8)	16·9 (2·4–42·5)
Malta	24·6 (4·4–59·4)	28·5 (15·9–38·1)	29·2 (12·2–44·2)	17·7 (2·7–44·2)
Monaco	27·1 (4·9–63·6)	27·0 (14·5–36·7)	29·1 (12·1–45·0)	16·8 (2·3–42·3)
Netherlands	27·6 (4·9–66·3)	26·7 (14·0–36·6)	29·1 (12·2–45·3)	16·6 (2·3–41·7)
Norway	27·6 (4·9–65·9)	26·7 (14·1–36·5)	29·1 (12·1–45·3)	16·7 (2·3–41·7)
Portugal	24·3 (4·3–58·4)	28·7 (15·9–38·5)	29·2 (12·2–44·1)	17·8 (2·8–44·1)
San Marino	26·8 (4·8–62·4)	27·2 (14·5–36·9)	29·1 (12·2–44·9)	16·9 (2·4–42·4)
Spain	27·0 (4·8–65·2)	27·0 (14·5–36·8)	29·1 (12·2–44·8)	16·8 (2·4–42·3)
Sweden	27·1 (4·8–64·5)	27·0 (14·5–36·8)	29·1 (12·2–44·9)	16·8 (2·4–42·1)
Switzerland	27·3 (4·8–64·5)	26·9 (14·5–36·7)	29·1 (12·2–45·2)	16·7 (2·4–42·2)
UK	25·2 (4·5–60·3)	28·1 (15·7–37·7)	29·2 (12·2–44·3)	17·5 (2·7–43·6)
**Latin America and Caribbean**	**9·1 (1·5–21·9)**	**38·1 (15·4–61·2)**	**29·7 (12·6–42·5)**	**23·1 (4·6–53·3)**
**Andean Latin America**	**7·8 (1·3–18·7)**	**38·9 (15·4–64·6)**	**29·7 (12·6–42·6)**	**23·5 (4·8–54·0)**
Bolivia	1·5 (0·0–7·1)	42·8 (14·9–77·7)	29·9 (12·6–43·3)	25·7 (5·5–57·7)
Ecuador	7·9 (1·3–18·8)	38·8 (15·4–64·7)	29·7 (12·6–42·8)	23·5 (4·7–54·1)
Peru	10·0 (1·7–24·2)	37·5 (15·6–59·6)	29·7 (12·7–42·5)	22·8 (4·4–52·5)
**Caribbean**	**7·8 (1·3–18·9)**	**38·9 (15·3–63·7)**	**29·7 (12·6–42·7)**	**23·5 (4·7–54·1)**
Antigua and Barbuda	10·9 (1·8–26·4)	37·0 (15·7–57·7)	29·6 (12·7–42·5)	22·4 (4·3–51·7)
The Bahamas	7·9 (1·2–19·2)	38·8 (15·4–64·0)	29·7 (12·6–42·5)	23·5 (4·8–54·1)
Barbados	11·9 (2·0–28·9)	36·4 (15·7–56·3)	29·6 (12·7–42·5)	22·1 (4·2–51·4)
Belize	5·2 (0·6–13·8)	40·5 (15·1–69·8)	29·8 (12·6–42·9)	24·5 (5·1–55·9)
Bermuda	20·6 (3·7–48·3)	31·0 (15·9–42·1)	29·3 (12·5–43·2)	19·1 (3·4–46·1)
Cuba	15·8 (2·7–37·4)	34·0 (15·6–48·6)	29·5 (12·9–42·5)	20·8 (3·8–47·8)
Dominica	4·6 (0·4–12·8)	40·9 (15·1–70·8)	29·8 (12·7–43·1)	24·7 (5·2–56·3)
Dominican Republic	5·4 (0·7–13·9)	40·4 (15·1–69·5)	29·8 (12·7–42·9)	24·4 (5·0–55·6)

Grenada	7·1 (1·1–17·5)	39·4 (15·4–65·7)	29·8 (12·6–42·7)	23·8 (4·9–54·3)
Guyana	1·5 (0·0–7·1)	42·8 (14·9–77·7)	29·9 (12·6–43·4)	25·7 (5·4–57·9)
Haiti	0·0 (0·0–0·0)	43·7 (14·8–80·7)	30·0 (12·6–43·6)	26·3 (5·6–59·2)
Jamaica	8·8 (1·4–21·3)	38·3 (15·4–61·9)	29·7 (12·6–42·6)	23·2 (4·6–53·7)
Puerto Rico	18·5 (3·3–44·2)	32·3 (15·8–44·7)	29·4 (12·9–42·7)	19·8 (3·6–46·8)
Saint Kitts and Nevis	12·2 (2·0–29·7)	36·2 (15·7–56·6)	29·6 (12·6–42·4)	22·0 (4·3–50·7)
Saint Lucia	8·5 (1·4–20·3)	38·5 (15·4–62·2)	29·7 (12·6–42·5)	23·3 (4·7–53·1)
Saint Vincent and the Grenadines	5·1 (0·6–13·5)	40·6 (15·2–69·8)	29·8 (12·7–42·9)	24·5 (5·1–55·8)
Suriname	3·2 (0·0–10·1)	41·8 (14·9–74·8)	29·9 (12·7–43·2)	25·2 (5·3–56·5)
Trinidad and Tobago	7·5 (1·2–18·4)	39·1 (15·4–64·8)	29·8 (12·6–42·5)	23·6 (4·8–54·3)
Virgin Islands	10·9 (1·9–26·2)	37·0 (15·7–57·4)	29·6 (12·7–42·5)	22·5 (4·3–52·2)
**Central Latin America**	**9·9 (1·7–23·9)**	**37·6 (15·5–59·7)**	**29·7 (12·6–42·5)**	**22·8 (4·5–52·5)**
Colombia	13·1 (2·2–31·4)	35·6 (15·6–53·5)	29·6 (12·8–42·5)	21·7 (4·1–50·6)
Costa Rica	15·3 (2·7–36·1)	34·2 (15·6–49·6)	29·5 (12·9–42·5)	20·9 (3·9–48·0)
El Salvador	9·3 (1·5–21·8)	38·0 (15·5–60·8)	29·7 (12·6–42·6)	23·0 (4·6–52·9)
Guatemala	2·7 (0·0–9·4)	42·0 (14·9–75·5)	29·9 (12·7–43·2)	25·3 (5·3–57·0)
Honduras	2·6 (0·0–9·4)	42·1 (14·9–75·3)	29·9 (12·6–43·2)	25·4 (5·4–57·0)
Mexico	9·7 (1·6–23·6)	37·7 (15·5–60·0)	29·7 (12·6–42·5)	22·9 (4·5–52·8)
Nicaragua	10·1 (1·7–24·5)	37·5 (15·5–59·0)	29·7 (12·6–42·5)	22·8 (4·4–52·6)
Panama	12·6 (2·2–29·8)	36·0 (15·5–54·4)	29·6 (12·7–42·5)	21·9 (4·2–51·0)
Venezuela	10·7 (1·8–26·0)	37·1 (15·7–58·6)	29·6 (12·7–42·5)	22·5 (4·4–52·3)
**Tropical Latin America**	**8·8 (1·4–20·9)**	**38·3 (15·4–61·9)**	**29·7 (12·6–42·6)**	**23·2 (4·6–53·9)**
Brazil	8·9 (1·4–21·0)	38·3 (15·4–61·9)	29·7 (12·6–42·5)	23·2 (4·6–53·8)
Paraguay	7·9 (1·2–19·0)	38·9 (15·4–63·8)	29·7 (12·6–42·6)	23·5 (4·7–54·5)
**North Africa and Middle East**	**9·6 (1·6–23·1)**	**37·8 (15·5–60·3)**	**29·7 (12·6–42·6)**	**22·9 (4·5–52·8)**
Afghanistan	0·0 (0·0–0·0)	43·7 (14·8–80·7)	30·0 (12·6–43·6)	26·3 (5·6–59·2)
Algeria	11·3 (1·9–27·3)	36·7 (15·6–56·9)	29·6 (12·7–42·4)	22·3 (4·3–51·4)
Bahrain	15·8 (2·8–37·8)	33·9 (15·6–48·3)	29·5 (12·9–42·6)	20·7 (3·8–47·9)
Egypt	6·6 (1·0–16·2)	39·7 (15·3–66·5)	29·8 (12·6–42·8)	24·0 (5·0–54·8)
Iran	13·9 (2·4–32·8)	35·1 (15·6–52·0)	29·5 (12·8–42·4)	21·4 (4·0–49·2)
Iraq	9·2 (1·5–21·7)	38·1 (15·5–61·3)	29·7 (12·6–42·6)	23·1 (4·6–53·3)
Jordan	13·9 (2·4–33·0)	35·1 (15·6–52·5)	29·5 (12·8–42·4)	21·4 (4·0–50·0)
Kuwait	20·1 (3·6–47·7)	31·3 (15·9–42·7)	29·4 (12·5–43·1)	19·3 (3·4–46·4)
Lebanon	19·3 (3·4–45·6)	31·8 (15·9–43·6)	29·4 (12·8–42·9)	19·5 (3·5–46·8)
Libya	9·8 (1·6–23·8)	37·7 (15·5–60·4)	29·7 (12·6–42·5)	22·8 (4·5–52·6)
Morocco	5·4 (0·7–14·3)	40·4 (15·1–69·4)	29·8 (12·7–42·8)	24·4 (5·0–55·1)
Oman	16·7 (2·9–39·2)	33·4 (15·6–47·4)	29·5 (12·9–42·6)	20·4 (3·7–47·8)
Palestine	9·9 (1·7–24·1)	37·6 (15·6–59·7)	29·7 (12·6–42·5)	22·8 (4·5–52·4)
Qatar	19·4 (3·4–46·2)	31·7 (15·9–43·4)	29·4 (12·7–42·9)	19·5 (3·5–46·5)
Saudi Arabia	15·7 (2·7–37·4)	34·0 (15·6–48·3)	29·5 (12·9–42·6)	20·8 (3·8–48·0)
Sudan	1·8 (0·0–7·6)	42·6 (14·9–77·6)	29·9 (12·6–43·2)	25·7 (5·5–57·4)
Syria	11·0 (1·8–27·2)	36·9 (15·7–57·5)	29·6 (12·7–42·5)	22·4 (4·3–51·8)
Tunisia	15·2 (2·6–35·9)	34·4 (15·6–49·6)	29·5 (12·9–42·5)	21·0 (3·9–48·3)
Turkey	15·3 (2·7–36·0)	34·3 (15·6–49·4)	29·5 (12·9–42·5)	20·9 (3·9–48·4)
United Arab Emirates	8·8 (1·5–21·3)	38·3 (15·4–62·0)	29·7 (12·6–42·6)	23·2 (4·6–53·3)
Yemen	0·6 (0·0–4·2)	43·4 (14·8–79·4)	30·0 (12·6–43·5)	26·1 (5·5–58·3)

**South Asia**	**0·3 (0·0–2·7)**	**43·5 (14·8–79·7)**	**30·0 (12·6–43·5)**	**26·1 (5·6–58·7)**
Bangladesh	1·4 (0·0–6·8)	42·9 (14·9–78·1)	29·9 (12·6–43·3)	25·8 (5·5–57·8)
Bhutan	2·8 (0·0–9·5)	42·0 (14·9–75·5)	29·9 (12·6–43·2)	25·3 (5·3–56·8)
India	0·3 (0·0–2·7)	43·6 (14·8–79·7)	30·0 (12·6–43·6)	26·2 (5·6–58·7)
Nepal	0·4 (0·0–3·4)	43·5 (14·8–79·4)	30·0 (12·6–43·5)	26·1 (5·5–58·6)
Pakistan	0·1 (0·0–0·6)	43·7 (14·8–80·5)	30·0 (12·6–43·6)	26·2 (5·6–59·2)
**Southeast Asia, east Asia, and Oceania**	**14·0 (2·4–33·1)**	**35·1 (15·6–51·4)**	**29·5 (12·8–42·5)**	**21·4 (4·0–49·4)**
**East Asia**	**18·5 (3·3–43·8)**	**32·3 (15·8–44·7)**	**29·4 (12·9–42·8)**	**19·8 (3·6–46·8)**
China	18·7 (3·3–44·2)	32·2 (15·8–44·4)	29·4 (12·8–42·9)	19·8 (3·6–46·8)
North Korea	5·4 (0·7–14·0)	40·4 (15·2–69·1)	29·8 (12·7–42·9)	24·4 (5·1–55·6)
Taiwan (province of China)	22·3 (4·0–53·5)	29·9 (16·0–40·4)	29·3 (12·3–43·5)	18·5 (3·2–45·5)
**Oceania**	**0·4 (0·0–1·3)**	**43·5 (14·8–79·8)**	**30·0 (12·5–43·4)**	**26·1 (5·6–59·0)**
American Samoa	4·2 (0·3–12·0)	41·1 (15·0–71·7)	29·9 (12·7–43·1)	24·8 (5·2–56·2)
Cook Islands	13·5 (2·2–34·4)	35·4 (15·8–53·3)	29·6 (12·7–42·6)	21·6 (3·9–50·0)
Fiji	1·0 (0·0–5·3)	43·1 (14·8–78·2)	30·0 (12·6–43·4)	25·9 (5·5–58·1)
Guam	9·3 (1·6–22·4)	38·0 (15·5–60·6)	29·7 (12·6–42·5)	23·0 (4·6–53·0)
Kiribati	0·0 (0·0–0·0)	43·7 (14·8–80·7)	30·0 (12·6–43·6)	26·3 (5·6–59·2)
Marshall Islands	0·1 (0·0–0·9)	43·7 (14·8–80·5)	30·0 (12·6–43·6)	26·2 (5·6–59·2)
Federated States of Micronesia	0·6 (0·0–4·4)	43·4 (14·8–78·8)	30·0 (12·6–43·4)	26·1 (5·5–58·3)
Nauru	4·0 (0·0–12·9)	41·3 (15·1–74·7)	29·9 (12·7–42·8)	24·9 (5·2–55·8)
Niue	10·5 (1·6–26·2)	37·3 (15·6–60·9)	29·7 (12·7–42·6)	22·6 (4·3–51·9)
Northern Mariana Islands	12·8 (2·2–30·4)	35·8 (15·6–53·6)	29·6 (12·7–42·5)	21·8 (4·1–50·0)
Palau	10·4 (1·5–27·5)	37·2 (15·7–59·7)	29·6 (12·5–42·5)	22·7 (4·2–52·1)
Papua New Guinea	0·0 (0·0–0·0)	43·7 (14·8–80·7)	30·0 (12·6–43·6)	26·3 (5·6–59·2)
Samoa	3·2 (0·0–10·5)	41·8 (15·0–74·7)	29·9 (12·7–43·2)	25·1 (5·3–56·5)
Solomon Islands	0·2 (0·0–2·2)	43·6 (14·8–80·2)	30·0 (12·6–43·6)	26·2 (5·6–59·1)
Tokelau	5·3 (0·0–16·6)	40·5 (15·3–69·9)	29·8 (12·7–42·9)	24·4 (4·9–55·4)
Tonga	2·5 (0·0–9·2)	42·2 (14·9–76·0)	29·9 (12·6–43·2)	25·4 (5·3–57·3)
Tuvalu	1·6 (0·0–8·7)	42·7 (15·0–77·6)	29·9 (12·7–43·4)	25·7 (5·4–57·5)
Vanuatu	0·0 (0·0–0·0)	43·7 (14·8–80·7)	30·0 (12·6–43·6)	26·2 (5·6–59·2)
**Southeast Asia**	**4·4 (0·7–11·4)**	**41·0 (15·1–71·4)**	**29·8 (12·7–43·1)**	**24·7 (5·1–56·5)**
Cambodia	0·9 (0·0–5·2)	43·2 (14·8–78·8)	30·0 (12·6–43·4)	26·0 (5·5–58·2)
Indonesia	1·0 (0·0–5·7)	43·2 (14·8–78·4)	30·0 (12·6–43·4)	25·9 (5·5–58·2)
Laos	0·1 (0·0–0·9)	43·7 (14·8–80·5)	30·0 (12·6–43·6)	26·2 (5·6–59·2)
Malaysia	11·1 (1·9–26·3)	36·9 (15·6–57·4)	29·6 (12·7–42·5)	22·4 (4·3–51·8)
Maldives	14·4 (2·5–34·4)	34·8 (15·6–51·1)	29·5 (12·8–42·5)	21·2 (3·9–49·7)
Mauritius	11·3 (1·9–28·0)	36·7 (15·7–56·4)	29·6 (12·7–42·5)	22·3 (4·2–51·7)
Myanmar	0·5 (0·0–4·3)	43·4 (14·8–79·4)	30·0 (12·6–43·5)	26·1 (5·5–58·5)
Philippines	1·5 (0·0–7·1)	42·8 (14·9–77·7)	29·9 (12·6–43·3)	25·7 (5·4–57·8)
Seychelles	9·0 (1·5–22·3)	38·1 (15·5–61·1)	29·7 (12·6–42·6)	23·1 (4·6–53·1)
Sri Lanka	13·3 (2·3–32·1)	35·5 (15·6–52·9)	29·6 (12·8–42·5)	21·6 (4·1–50·0)
Thailand	13·1 (2·2–31·3)	35·6 (15·5–53·4)	29·6 (12·8–42·5)	21·7 (4·1–50·2)
Timor-Leste	0·2 (0·0–2·3)	43·6 (14·8–79·7)	30·0 (12·6–43·6)	26·2 (5·5–59·0)
Vietnam	9·8 (1·7–23·8)	37·7 (15·5–60·1)	29·7 (12·6–42·5)	22·8 (4·5–52·8)
**Sub-Saharan Africa**	**0·1 (0·0–0·6)**	**43·7 (14·8–80·4)**	**30·0 (12·6–43·5)**	**26·2 (5·6–59·2)**
**Central sub-Saharan Africa**	**0·0 (0·0–0·1)**	**43·7 (14·8–80·7)**	**30·0 (12·6–43·6)**	**26·3 (5·6–59·2)**

Angola	0·0 (0·0–0·0)	43·7 (14·8–80·7)	30·0 (12·6–43·6)	26·3 (5·6–59·2)
Central African Republic	0·0 (0·0–0·0)	43·7 (14·8–80·7)	30·0 (12·6–43·6)	26·3 (5·6–59·2)
Congo (Brazzaville)	0·0 (0·0–0·0)	43·7 (14·8–80·7)	30·0 (12·6–43·6)	26·3 (5·6–59·2)
DR Congo	0·0 (0·0–0·0)	43·7 (14·8–80·7)	30·0 (12·6–43·6)	26·3 (5·6–59·2)
Equatorial Guinea	0·5 (0·0–4·0)	43·4 (14·8–79·4)	30·0 (12·6–43·5)	26·1 (5·5–58·2)
Gabon	0·3 (0·0–3·1)	43·6 (14·8–79·7)	30·0 (12·6–43·6)	26·2 (5·5–58·6)
**Eastern sub-Saharan Africa**	**0·0 (0·0–0·1)**	**43·7 (14·8–80·7)**	**30·0 (12·6–43·6)**	**26·2 (5·6–59·2)**
Burundi	0·0 (0·0–0·0)	43·7 (14·8–80·7)	30·0 (12·6–43·6)	26·3 (5·6–59·2)
Comoros	0·0 (0·0–0·0)	43·7 (14·8–80·7)	30·0 (12·6–43·6)	26·3 (5·6–59·2)
Djibouti	0·1 (0·0–0·9)	43·7 (14·8–80·5)	30·0 (12·6–43·6)	26·2 (5·6–59·2)
Eritrea	0·0 (0·0–0·0)	43·7 (14·8–80·7)	30·0 (12·6–43·6)	26·3 (5·6–59·2)
Ethiopia	0·0 (0·0–0·0)	43·7 (14·8–80·7)	30·0 (12·6–43·6)	26·3 (5·6–59·2)
Kenya	0·0 (0·0–0·1)	43·7 (14·8–80·7)	30·0 (12·6–43·6)	26·2 (5·6–59·2)
Madagascar	0·0 (0·0–0·0)	43·7 (14·8–80·7)	30·0 (12·6–43·6)	26·3 (5·6–59·2)
Malawi	0·0 (0·0–0·0)	43·7 (14·8–80·7)	30·0 (12·6–43·6)	26·3 (5·6–59·2)
Mozambique	0·0 (0·0–0·0)	43·7 (14·8–80·7)	30·0 (12·6–43·6)	26·3 (5·6–59·2)
Rwanda	0·0 (0·0–0·1)	43·7 (14·8–80·5)	30·0 (12·6–43·6)	26·2 (5·6–59·2)
Somalia	0·0 (0·0–0·0)	43·7 (14·8–80·7)	30·0 (12·6–43·6)	26·3 (5·6–59·2)
South Sudan	0·0 (0·0–0·0)	43·7 (14·8–80·7)	30·0 (12·6–43·6)	26·3 (5·6–59·2)
Uganda	0·0 (0·0–0·0)	43·7 (14·8–80·7)	30·0 (12·6–43·6)	26·2 (5·6–59·2)
Tanzania	0·0 (0·0–0·0)	43·7 (14·8–80·7)	30·0 (12·6–43·6)	26·2 (5·6–59·2)
Zambia	0·1 (0·0–0·5)	43·7 (14·8–80·5)	30·0 (12·6–43·6)	26·2 (5·6–59·2)
**Southern sub-Saharan Africa**	**1·3 (0·0–5·8)**	**42·9 (14·9–78·1)**	**30·0 (12·5–43·3)**	**25·8 (5·5–57·8)**
Botswana	3·4 (0·0–10·5)	41·6 (15·0–73·4)	29·9 (12·7–43·2)	25·1 (5·3–56·4)
eSwatini	0·1 (0·0–0·9)	43·7 (14·8–80·5)	30·0 (12·6–43·6)	26·2 (5·6–59·2)
Lesotho	0·0 (0·0–0·0)	43·7 (14·8–80·7)	30·0 (12·6–43·6)	26·3 (5·6–59·2)
Namibia	1·0 (0·0–5·6)	43·1 (14·9–78·4)	30·0 (12·6–43·4)	25·9 (5·5–58·2)
South Africa	1·7 (0·0–7·6)	42·7 (14·9–78·1)	29·9 (12·6–43·2)	25·7 (5·4–57·8)
Zimbabwe	0·0 (0·0–0·0)	43·7 (14·8–80·7)	30·0 (12·6–43·6)	26·3 (5·6–59·2)
**Western sub-Saharan Africa**	**0·1 (0·0–0·4)**	**43·7 (14·8–80·6)**	**30·0 (12·6–43·6)**	**26·2 (5·6–59·2)**
Benin	0·0 (0·0–0·0)	43·7 (14·8–80·7)	30·0 (12·6–43·6)	26·3 (5·6–59·2)
Burkina Faso	0·0 (0·0–0·0)	43·7 (14·8–80·7)	30·0 (12·6–43·6)	26·3 (5·6–59·2)
Cape Verde	6·3 (0·9–15·9)	39·9 (15·3–67·7)	29·8 (12·6–42·8)	24·1 (5·0–55·2)
Cameroon	0·1 (0·0–0·5)	43·7 (14·8–80·5)	30·0 (12·6–43·6)	26·2 (5·6–59·2)
Chad	0·0 (0·0–0·0)	43·7 (14·8–80·7)	30·0 (12·6–43·6)	26·3 (5·6–59·2)
Côte d'Ivoire	0·0 (0·0–0·0)	43·7 (14·8–80·7)	30·0 (12·6–43·6)	26·3 (5·6–59·2)
The Gambia	0·0 (0·0–0·0)	43·7 (14·8–80·7)	30·0 (12·6–43·6)	26·2 (5·6–59·2)
Ghana	0·2 (0·0–2·3)	43·6 (14·8–80·2)	30·0 (12·6–43·6)	26·2 (5·6–59·1)
Guinea	0·0 (0·0–0·0)	43·7 (14·8–80·7)	30·0 (12·6–43·6)	26·3 (5·6–59·2)
Guinea-Bissau	0·0 (0·0–0·0)	43·7 (14·8–80·7)	30·0 (12·6–43·6)	26·3 (5·6–59·2)
Liberia	0·0 (0·0–0·0)	43·7 (14·8–80·7)	30·0 (12·6–43·6)	26·3 (5·6–59·2)
Mali	0·0 (0·0–0·0)	43·7 (14·8–80·7)	30·0 (12·6–43·6)	26·3 (5·6–59·2)
Mauritania	0·3 (0·0–2·5)	43·6 (14·8–79·7)	30·0 (12·6–43·6)	26·2 (5·6–58·9)
Niger	0·0 (0·0–0·0)	43·7 (14·8–80·7)	30·0 (12·6–43·6)	26·3 (5·6–59·2)
Nigeria	0·0 (0·0–0·4)	43·7 (14·8–80·5)	30·0 (12·6–43·6)	26·2 (5·6–59·2)
São Tomé and PrÍncipe	0·5 (0·0–4·1)	43·4 (14·8–79·4)	30·0 (12·6–43·4)	26·1 (5·5–58·3)

Senegal	0·0 (0·0–0·0)	43·7 (14·8–80·7)	30·0 (12·6–43·6)	26·3 (5·6–59·2)
Sierra Leone	0·0 (0·0–0·0)	43·7 (14·8–80·7)	30·0 (12·6–43·6)	26·3 (5·6–59·2)
Togo	0·0 (0·0–0·0)	43·7 (14·8–80·7)	30·0 (12·6–43·6)	26·2 (5·6–59·2)

Numbers in parentheses represent 95% uncertainty intervals.

References

1. Hetrick SE, Purcell R, Garner B, Parslow R. Combined pharmacotherapy and psychological therapies for post traumatic stress disorder (PTSD). *Cochrane Database of Systematic Reviews* 2010; (7).
2. Ori R, Amos T, Bergman H, Soares-Weiser K, Ipser JC, Stein DJ. Augmentation of cognitive and behavioural therapies (CBT) with d-cycloserine for anxiety and related disorders. *Cochrane Database of Systematic Reviews* 2015; (5).
3. Abbass AA, Kisely SR, Town JM, et al. Short-term psychodynamic psychotherapies for common mental disorders. *Cochrane Database of Systematic Reviews* 2014; (7).
4. Depping AM, Komossa K, Kissling W, Leucht S. Second-generation antipsychotics for anxiety disorders. *Cochrane Database of Systematic Reviews* 2010; (12).
5. Thorlund K, Mills E. Stability of additive treatment effects in multiple treatment comparison meta-analysis: a simulation study. *Clin Epidemiol* 2012; **4**: 75-85.
6. Mills EJ, Thorlund K, Ioannidis JPA. Calculating additive treatment effects from multiple randomized trials provides useful estimates of combination therapies. *Journal of Clinical Epidemiology* 2012; **65**(12): 1282-8.
7. Ware J, Jr., Kosinski M, Keller SD. A 12-Item Short-Form Health Survey: construction of scales and preliminary tests of reliability and validity. *Med Care* 1996; **34**(3): 220-33.
8. Zheng P, Barber R, Sorensen RJD, Murray CJL, Aravkin AY. Trimmed Constrained Mixed Effects Models: Formulations and Algorithms. *Journal of Computational and Graphical Statistics* 2021.
9. Alonso J, Liu Z, Evans-Lacko S, et al. Treatment gap for anxiety disorders is global: Results of the World Mental Health Surveys in 21 countries. *Depress Anxiety* 2018; **35**(3): 195-208.
